# Recent Advances on Transition Metal Sulfides for Key Components of Lithium–Sulfur Batteries

**DOI:** 10.1002/advs.202512191

**Published:** 2025-09-14

**Authors:** Jingxuan Chen, Tingting Sun, Junliang Liu, Hao Yu, Qisheng Zang, Tong Yuan, Fuqin Zhang

**Affiliations:** ^1^ State Key Laboratory of Powder Metallurgy Central South University Changsha 410083 P. R. China

**Keywords:** anode, cathode, Li–S batteries, separator, shuttle effect, TMSs

## Abstract

Lithium–sulfur batteries (LSBs), renowned for their cost‐effectiveness and high energy density, stand out as highly promising energy storage solutions with substantial application potential. Nevertheless, the shuttle effect—caused by the migration of polysulfides between cathode and anode—severely hampers their practical implementation. Extensive research has demonstrated that transition metal sulfide (TMS) catalysts can effectively mitigate this shuttle phenomenon. In LSBs, the cathode, anode, and separator represent pivotal components, and their properties play a decisive role in determining the electrochemical performance. As such, current mainstream modification strategies mainly target these three key areas. Therefore, this review focuses on the latest advances in the application of TMSs in key components of LSBs. It systematically analyzes the core issues currently faced, deeply elaborates on the roles and shortcomings of different TMS modification methods and their application approaches in various LSB components, and discusses the underlying mechanisms. Aiming at the above problems, this paper proposes potential solutions and research directions, hoping to provide references for researchers in related fields.

## Graphical abstract

This review systematically summarizes the latest advances in the application of transition metal sulfide catalysts in three key components of lithium–sulfur batteries (LSBs). Specifically, these catalysts can function respectively as sulfur host materials, separator coatings, and lithium anode stabilizers. By mitigating the shuttle effect and lithium dendrite growth, they ultimately enable a marked enhancement in the comprehensive performance of LSBs.

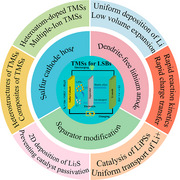

## 1 Introduction

**Figure 1:**
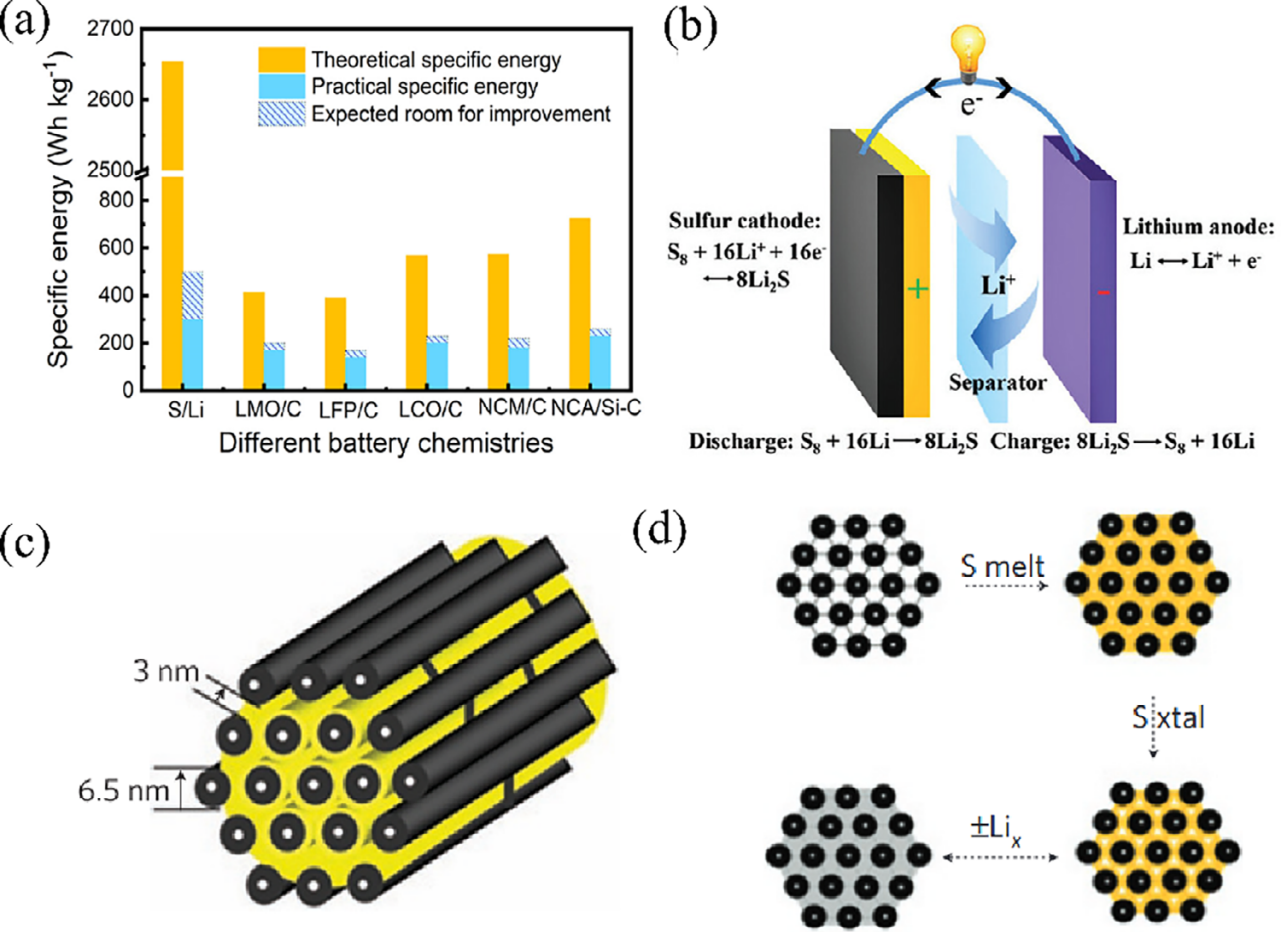
a) Theoretical and practical specific energy of various battery chemistries. Reproduced with permission.^[^ ^12^ ^]^ Copyright 2020, Elsevier. b) Schematic of the electrochemistry of LSBs in ether‐based electrolytes. Reproduced with permission.^[^ ^18^ ^]^ Copyright 2016, Royal Society of Chemistry. c) A schematic diagram of the sulfur (yellow) confined in the interconnected pore structure of mesoporous carbon, CMK‐3. Reproduced with permission.^[^ ^21^ ^]^ Copyright 2009, Springer Nature. d) Schematic diagram of composite synthesis by impregnation of molten sulfur. Reproduced with permission.^[^ ^21^ ^]^ Copyright 2009, Springer Nature.

The research and application of energy storage devices play a crucial role in promoting the popularization of new energy and solving the energy crisis.^[^ ^1^, ^2^ ^]^ However, with the progress of society and technology, the performance of commercial lithium‐ion batteries has approached the theoretical energy density limit (420 Wh kg^−1^), which makes it challenging to meet the working requirements under extreme conditions.^[^ ^3^, ^4^, ^5^, ^6^ ^]^ In addition, their low specific capacity and energy density are difficult to meet the requirements of large equipment such as aircraft, ships, and electric vehicles, thus limiting the further development of lithium‐ion batteries as energy storage devices and propelling the development of new‐generation lithium batteries.^[^ ^7^ ^]^ In the 1960s, lithium–sulfur batteries (LSBs) were first proposed by Herbert and Ulam.^[^ ^8^ ^]^ Endowed with a high theoretical energy density of 2600 Wh kg^−1^ and a theoretical specific capacity of 1675 mAh g^−1^, LSBs exhibit values ≈5–7 times higher than those of lithium‐ion batteries (**Figure** **1**a).^[^ ^9^, ^10^, ^11^, ^12^ ^]^ Moreover, the abundant reserves and low cost of sulfur are also advantages.^[^ ^13^ ^]^ Hence, LSBs are regarded as the most prospective next‐generation energy storage devices. Their working principle is different from that of lithium‐ion batteries, which involves the process of lithium‐ion insertion/extraction. LSBs are charged and discharged through oxidation‐reduction reactions.^[^ ^14^ ^]^ The active materials of the cathode and anode are sulfur (S_8_) and lithium metal, respectively, which are separated by a separator in the middle to prevent a short circuit.^[^ ^13^, ^15^ ^]^ After the organic electrolyte is added, the total reaction formula is as follows: S_8_ + 16 Li^+^ + 16 e^−^ → 8 Li_2_S (Figure 1b).^[^ ^16^, ^17^, ^18^ ^]^ The reaction process involves the transfer of 16 electrons, which guarantees that the battery has an ultra‐high theoretical specific capacity. However, in the actual sulfur redox reaction, affected by the intrinsic defects such as poor conductivity of sulfur cathode, unstable activity of lithium anode, and high solubility of polysulfides in electrolyte, the practical performance of LSBs is significantly deteriorated, accompanied by multiple issues including shuttle effect, lithium dendrite growth, and sluggish reaction kinetics.^[^ ^19^, ^20^ ^]^

Among them, the shuttle effect of polysulfides represents a core bottleneck issue, and suppressing the generation and evolution of this effect serves as a key technical pathway to enhance battery performance. In 2009, the Nazar team first used the melting method to combine sulfur with mesoporous carbon (CMK‐3) to form a composite material as the cathode of LSBs (Figure 1c).^[^ ^21^ ^]^ Due to the confinement of sulfur in the mesopores of the composite material, the dissolution of polysulfides in the electrolyte was reduced, which suppressed the shuttle effect to a certain degree and greatly improved the discharge capacity and cycling performance (Figure 1d). Since then, developing sulfur host materials compatible with cathodes to fundamentally suppress the shuttle effect has become a core research direction in the field of LSBs. However, studies indicate that carbon/sulfur composites, despite suppressing the shuttle effect and improving cycle performance, exhibit weak redox catalysis that hampers sulfur reaction kinetics. To address this challenge, investigators have developed multiple polar materials such as transition metal oxides (TMOs),^[^ ^22^ ^]^ transition metal sulfides (TMSs),^[^ ^23^ ^]^ transition metal nitrides (TMNs),^[^ ^24^ ^]^ transition metal phosphides (TMPs),^[^ ^25^ ^]^ single‐atom catalysts, transition metal carbides/nitrides/carbonitrides (MXenes), and metal organic frameworks (MOFs).^[^ ^26^ ^]^ After comprehensive consideration, TMSs exhibit great application potential in LSBs and outshine other transition metal compounds with a plethora of merits, such as remarkable electrical conductivity, cost‐effectiveness, structural robustness, and facile synthesis methods.^[^ ^27^, ^28^ ^]^ However, follow‐up studies have shown that modifying sulfur cathodes alone is insufficient to completely suppress the shuttling of polysulfides between the electrodes, ultimately leading to battery failure.^[^ ^29^, ^30^, ^31^ ^]^ Additionally, the separator not only isolates electron transport to prevent battery short‐circuiting but also blocks the diffusion path of polysulfides, thus effectively inhibiting the shuttling effect. Meanwhile, the stability of the lithium anode is equally critical for battery safety.^[^ ^32^ ^]^ In recent years, TMSs have demonstrated versatile applications in the energy storage field, particularly excelling in cathodes, anodes, and separators, thanks to their unique layered structures, exceptional electrocatalytic activity, and semiconductor properties. For instance, when CoNi_2_S_4_ hollow nanospheres are used as cathodes in magnesium‐ion batteries, they exhibit a higher specific surface area, lower impedance, and faster magnesium‐ion diffusion rate.^[^ ^33^ ^]^ Furthermore, TMS coatings applied to separators not only help maintain the structural stability of the cathode and anode but also accelerate ion transport efficiency. Eunho et al.^[^ ^34^ ^]^ uniformly coated MoS_2_ nanolayers on the surface of lithium metal as a protective layer, which effectively inhibits dendrite growth, more than doubling the cycle life of lithium symmetric batteries while laying the foundation for the construction of lithium‐based metal batteries with higher energy density. In LSBs, TMSs have been widely studied as catalytic materials, demonstrating remarkable advantages in enhancing electronic conductivity, optimizing reaction kinetics, and suppressing the shuttle effect, which has prompted extensive research focus on the development of efficient TMS catalysts. It is noteworthy that the performance of three core components—the cathode, separator, and anode—directly determines the overall performance of LSBs. The application of TMSs in these three components can specifically address their inherent defects, thereby improving the comprehensive battery performance. Based on this, this review focuses on transition metal sulfide catalysts, highlighting their unique advantages over other polar materials. Next, it systematically combs the latest research progress, elaborates on their application scenarios and action mechanisms in the cathode, separator, and anode, and analyzes typical research results, aiming to provide systematic references for researchers in the field.

## 2 TMS Catalysts for the Sulfur Host Cathode

### 2.1 The Advantages and Disadvantages of TMSs for the Sulfur Host Cathode

By virtue of their unique structural advantages and chemical properties, TMSs were first employed in the intensively studied sulfur host cathodes. TMSs typically exist as 2D layered materials. The layers are stacked through Van Der Waals forces, giving rise to abundant inter‐layer voids. This graphene‐like architecture endows them with robust sulfur‐storage capabilities. It cannot only effectively cope with the substantial volume variation of the sulfur cathode during the pre‐reaction and post‐reaction processes but also guarantee the swift conduction of electrons.^[^ ^35^ ^]^ Consequently, in comparison with other materials, they stands out as an outstanding option for sulfur host materials. Meanwhile, TMSs, as polar materials, interact with polysulfides through robust physical and chemical adsorption. Additionally, metal sulfides exhibit remarkable affinity for sulfur.^[^ ^36^ ^]^ Their metallic constituents can form chemical bonds with the sulfur atoms in polysulfides, while the sulfur moieties can bond with lithium ions. Besides the adsorption function, TMSs showcase catalytic activity, facilitating the transformation of liquid polysulfides into solid lithium sulfide, thus effectively inhibiting the shuttle effect. Regarding electrical conductivity, the layered structure of sulfides furnishes suitable pathways for electron transfer. In comparison to polar materials such as oxides, carbides, selenides, nitrides, and phosphides, sulfides exhibit superior electrical conductivity and more rapid ion‐transport capabilities.^[^ ^37^ ^]^ During the course of the battery reaction, sulfides are capable of enhancing the electrical conductivity of the sulfur cathode, thereby facilitating electron transfer. Moreover, sulfides demonstrate a robust chemical affinity toward LiPSs and possess an abundance of active sites, which in turn accelerate the redox reaction kinetics.^[^ ^35^ ^]^ Consequently, sulfides hold substantial application potential in augmenting the performance of LSBs and propelling the advancement of energy storage. When transition metal sulfides are employed as sulfur host cathode in Li–S batteries, the following criteria must be satisfied^[^ ^38^, ^39^, ^40^, ^41^ ^]^: 1) The host material ought to exhibit suitable pore sizes. Subsequent to the formation of a composite with sulfur via the melt‐infiltration technique, the cathode is enabled to reach a higher sulfur content, consequently allowing for the attainment of a higher energy density and discharge capacity. Experimental results reveal that the pore‐size range should lie between the pore size of micropores (0.6–2 nm) and that of mesopores (2–50 nm). 2) The host material is required to exhibit high electrical conductivity. This inherent property ensures that a greater quantity of active substances partake in the reaction, thereby enhancing the specific discharge capacity of the actual battery. Moreover, it can also facilitate the kinetics of the redox reaction and mitigate the degree of polarization. 3) The host material must possess robust adsorption and catalytic capabilities. Only by virtue of such capabilities can it interact with the liquid‐phase polysulfides, inhibit the shuttle effect, and thereby enhance the long‐cycle stability and rate performance of Li–S cells.

The properties of typical TMS catalysts are summarized in **Table** **1**, from which the distinct advantages of different TMSs can be clearly identified, thereby enabling the selection of suitable catalytic materials and their corresponding modification methods to be facilitated.

**Table 1:** Comparison of properties of several typical TMSs.

TMSs	Structure	Conductivity [S m^−1^]	Adsorption energy for LiPSs [eV]	Catalytic ability	Catalytic mechanism	Refs.
MoS_2_	Hexagonal layered structure	10^0^–10^4^	−1.2—2.5	Moderate activity	Dangling bonds toward the adsorption and conversion of LiPSs	[42]
WS_2_	Hexagonal layered structure	10^0^–10^2^	−1.5—2.8	Moderate activity	Dangling bonds toward the adsorption and conversion of LiPSs	[42]
CoS_2_	Pyrite‐type cubic structure	10^4^–10^5^	−2.3—3.0	High activity	Multivalent atoms tend to participate in electron transfer (Co^2+^/Co^3+^)	[43]
NiS_2_	Pyrite‐type cubic structure	10^4^	−1.9—2.8	High activity	Multivalent atoms tend to participate in electron transfer (Ni^2+^/Ni^3+^)	[44]
FeS_2_	Pyrite‐type cubic structure	10^2^–10^4^	−1.1—1.9	Moderate activity	Multivalent atoms tend to participate in electron transfer (Fe^2+^/Fe^3+^)	[45]
VS_2_	Hexagonal layered structure	10^4^–10^5^	−1.6–−2.2	High activity	Multivalent atoms tend to participate in electron transfer (V^3+^/V^4+^)	[42]
ZnS	Cubic zinc blende	10^−14^	−2.0—3.2	Low activity	Physical adsorption and weak activation	[46]
SnS_2_	Hexagonal layered structure	10^−4^–10^−2^	−1.5—2.5	Low activity	Physical adsorption and weak activation	[47]
CoNi_2_S_4_	Spinel cubic structure	10^2^–10^4^	−1.2—3.7	High activity	Dual‐site synergistic effect and abundant active sites	[48]

**Figure 2:**
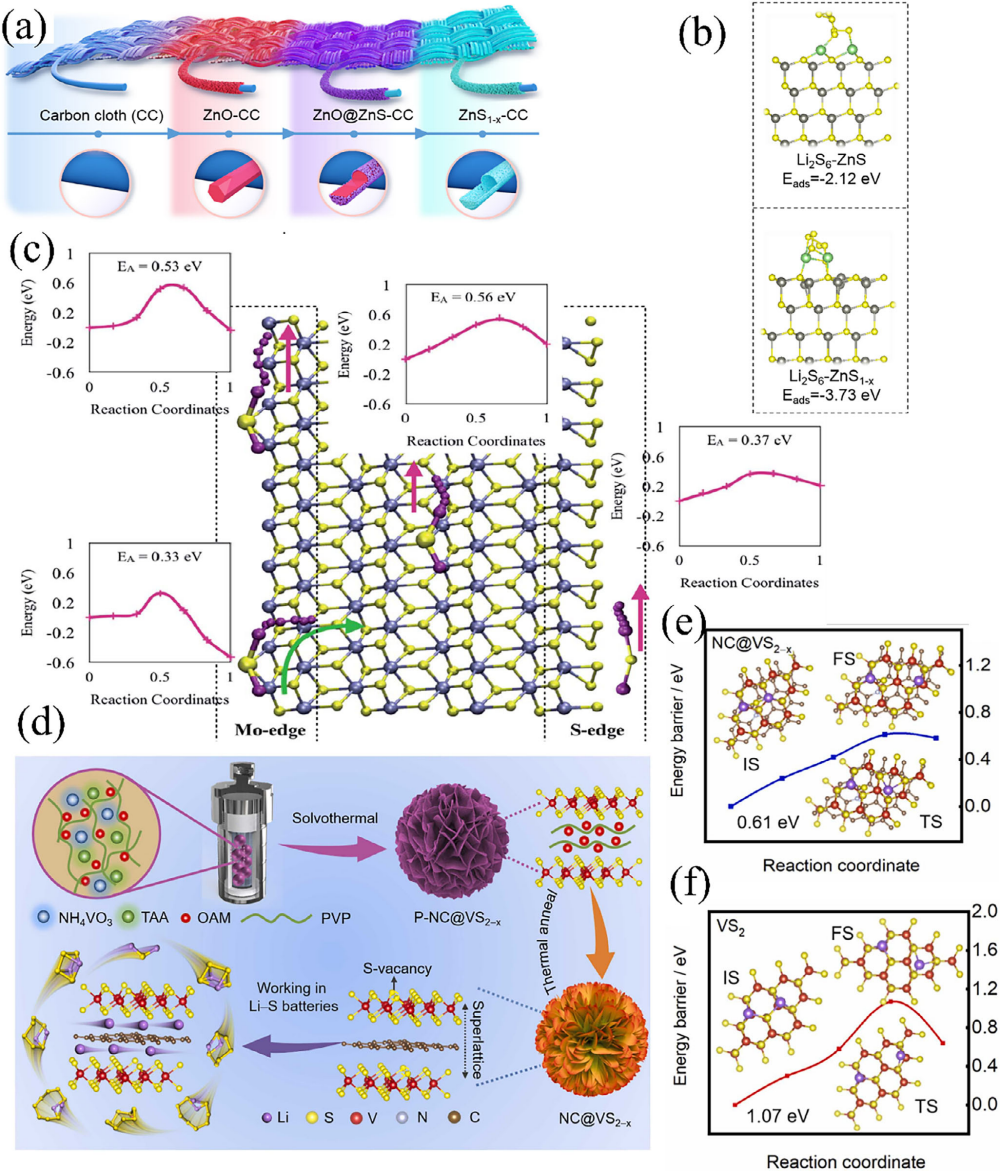
a) Schematic illustrating the synthesis process of the sulfur deficient ZnS_1−x_ coated carbon cloth. Reproduced with permission.^[^ ^50^ ^]^ Copyright 2021, Elsevier. b) The optimized geometrical configurations of Li_2_S_6_‐ZnS and Li_2_S_6_‐ZnS_1−x_. Reproduced with permission.^[^ ^50^ ^]^ Copyright 2021, Elsevier. c) First‐principle calculations of the electrochemical dissociation paths of Li_2_S at various sites on 1T MoS_2_. Reproduced with permission.^[^ ^51^ ^]^ Copyright 2020, Royal Society of Chemistry. d) Schematic illustration of the synthetic procedures for NC@VS_2–x_ and the role of NC@VS_2–x_ played in sulfur chemistry. Reproduced with permission.^[^ ^52^ ^]^ Copyright 2023, Elsevier. e) Li_2_S decomposition energy barriers on NC@VS_2–x_ and f) VS_2_ matrix. Reproduced with permission.^[^ ^52^ ^]^ Copyright 2023, Elsevier.

With the growing application and research of transition metal compounds in LSBs, results have shown that transition metal atoms can bind with sulfur to form stable metal‐sulfur bonds, which can adsorb and simultaneously promote the conversion of polysulfides and the decomposition of lithium sulfide.^[^ ^49^ ^]^ Drawing on this, sulfides of corresponding transition metal atoms were subsequently also used as cathode materials. For example, Wang et al.^[^ ^50^ ^]^ deposited ZnO onto commercial carbon cloth and fabricated the ZnS_1‐x_‐CC composite with sulfur vacancies via high‐temperature vulcanization and acetic‐acid etching procedures (**Figure** **2**a). This material features a tubular morphology and is homogeneously dispersed on the carbon cloth, thereby furnishing a profusion of active sites for the sulfur–involved electrochemical reactions. Additionally, its copious sulfur‐vacancy defects augment the adsorption ability toward polysulfides. Through density functional theory (DFT) calculations, it is found that the binding energy of ZnS_1‐x_ with sulfur vacancies to Li_2_S_6_ amounts to −3.73 eV (Figure 2b), which is markedly higher than that of ZnS to Li_2_S_6_ (−2.12 eV). The existence of vacancies elevates both the catalytic capability and the velocity of electron transfer. Consequently, this propels the conversion and decomposition of polysulfides while concurrently suppressing the shuttle effect. At a current density of 1 C, the Li_2_S_6_/ZnS_1‐x_‐CC cathode exhibited an original specific capacity of 659 mAh g^−1^. Following 500 charge–discharge cycles, the specific capacity was preserved at 524 mAh g^−1^, and the average decay rate was as low as 0.04%. Compared with carbon‐based sulfur hosts, this catalyst demonstrates stronger adsorption and catalytic capabilities, and thus plays a more significant role in enhancing the performance of LSBs.

Single‐component MoS_2_ has also found applications in Li–S batteries. Xu et al.^[^ ^51^ ^]^ revealed via first‐principles calculations that, in contrast to the 2H phase, the surface and edge Mo sites of the 1T‐phase MoS_2_ exhibit more robust adsorption and catalytic capabilities. The binding energies of the 1T and 2H phases to Li_2_S are 3.77 and 1.46 eV, respectively. Figure 2c depicts the molecular structure of the 1T‐phase MoS_2_ and the potential energy surface for Li_2_S dissociation at different sites (Mo edge sites and S edge sites). Evidently, within the 1T phase, the energy barrier for Li_2_S decomposition is lower. Furthermore, the length of the Li–S bond expands from 2.1 Å in a vacuum environment to 2.55 and 2.65 Å within the 2H and 1T phases, respectively. These findings collectively validate that the 1T‐phase MoS_2_ exhibits more potent catalytic capabilities and is expected to yield superior performance when utilized in lithium‐sulfur cells. Subsequently, the 1T‐phase MoS_2_ was synthesized via ball‐milling in combination with n‐butyllithium intercalation. It was then incorporated with porous carbon materials to fabricate an electrode for performance assessment. With a sulfur content as high as 81 wt.% and a current density of 0.05 C, the initial specific discharge capacity attained 856 mAh g^−1^. After 300 charge–discharge cycles, the specific capacity could still be maintained above 600 mAh g^−1^, signifying a high utilization efficiency of active substances and excellent reversibility. Similarly, Qin et al.^[^ ^52^ ^]^ proposed superlattice and sulfur‐vacancy engineering to enhance the adsorption and catalytic capabilities of sulfides. A composite material, in which VS_2_ with both superlattice and sulfur vacancies is embedded in N‐doped carbon (NC@VS_2‐x_), was constructed for the catalytic conversion of LiPSs (Figure 2d). Among them, the superlattice embedded in NC and the defect engineering generated highly active trapping sites, enhanced the electronic conductivity, and provided rapid lithium‐ion diffusion channels. This enabled a large amount of Li_2_S to precipitate, promoted the dissolution of solid sulfur species, and accelerated the lithium‐ion transport. In addition, during the discharge process, the decomposition energy barrier of Li_2_S undergoes a remarkable reduction, dropping from 1.07 eV on the VS_2_ matrix (Figure 2e) to 0.61 eV on the NC@VS_2‐x_ substrate (Figure 2f). This substantial decline effectively mitigates the polarization of the battery to a great extent. In the case where the sulfur loading reached 6.2 mg cm^−2^ and the lean E/S ratio was maintained at 4.0 µL mg^−1^, the area capacity of the NC@VS_2‐x_ battery reached 5.15 mAh cm^−2^.

However, the majority of pure‐component metal sulfides exist in the semiconductor phase. This characteristic restricts the electrical conductivity of these materials to a certain degree, rendering it arduous for them to satisfy the demands in specific situations. Moreover, their high crystallinity confines the catalytic action on polysulfides to merely the edge sites and a limited number of dangling‐bond‐adjacent regions. As a result, the catalytic ability of metal sulfides is substantially compromised. Additionally, an excessively strong adsorption capacity also impedes the subsequent catalysis of reaction products.^[^ ^53^, ^54^ ^]^ Consequently, achieving a balance between the adsorption and catalytic capabilities is of equal significance. Therefore, modifying the sulfides themselves to enhance their electrical conductivity, adsorption capacity, and catalytic performance comprehensively represents a current research focus and a pivotal step in propelling the commercialization of Li–S cells. Mainstream TMS modification methods currently fall into four categories: heteroatom doping, polymetallic sulfides, heterostructures, and composites. TMSs modified through distinct strategies each exhibit unique positive effects in the cathode, yet they also have inherent limitations. Subsequent sections will elaborate on these four modification strategies sequentially, covering their operating mechanisms in LBSs, impacts on reaction kinetics, and associated performance enhancements. This aims to fully clarify the application pathways of TMSs in LSBs as well as future avenues for improvement.

### 2.2 Heteroatom Doping Modification of TMSs

#### 2.2.1 Increase the Number of Active Sites

**Figure 3:**
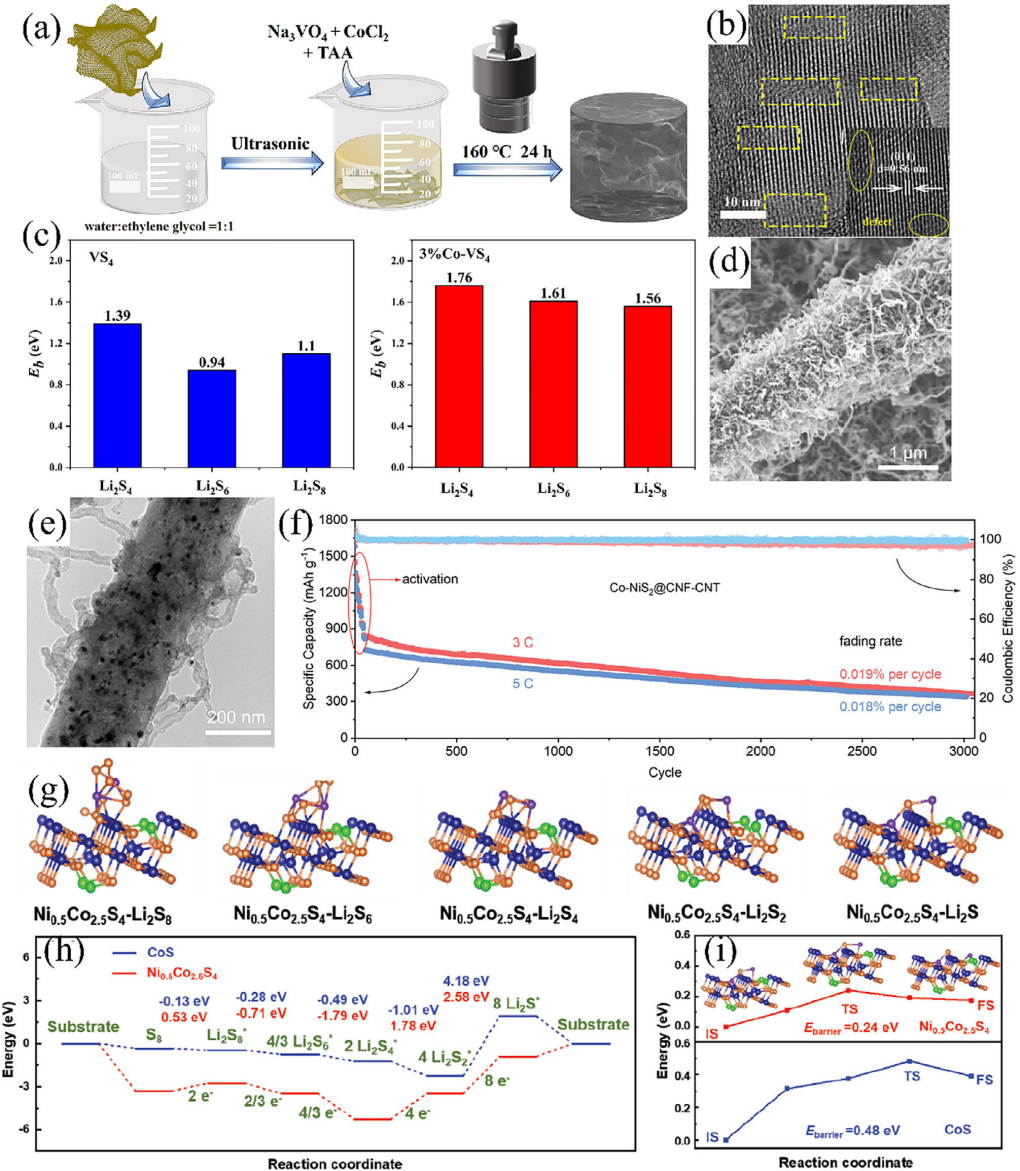
a) Fabrication Route of x%Co‐VS_4_/rGO (x: the Molar Content of Cobalt) Composites. Reproduced with permission.^[^ ^56^ ^]^ Copyright 2022, American Chemical Society. b) HR‐TEM image of 3%Co‐VS_4_/rGO and the lattice spacing at the selected area. Reproduced with permission.^[^ ^56^ ^]^ Copyright 2022, American Chemical Society. c) Binding energies (Eb) for Li_2_S_x_ on VS_4_ and 3%Co‐VS_4_. Reproduced with permission.^[^ ^56^ ^]^ Copyright 2022, American Chemical Society. d) SEM image and e) TEM image of Co‐NiS_2_@CNF‐CNT. Reproduced with permission.^[^ ^57^ ^]^ Copyright 2023, Wiley‐VCH. f) Long cycle life tests of Li–S batteries with Co‐NiS_2_@CNF‐CNT at 3 and 5 C for 3000 cycles. Reproduced with permission.^[^ ^57^ ^]^ Copyright 2023, Wiley‐VCH. g) The optimized structure configurations of Li_2_S_8_, Li_2_S_6_, Li_2_S_4_, Li_2_S_2_, and Li_2_S on the Ni_0.5_Co_2.5_S_4_ surface. Reproduced with permission.^[^ ^58^ ^]^ Copyright 2024, Wiley‐VCH. h) Energy profiles for the SRR on the CoS and Ni_0.5_Co_2.5_S_4_ surfaces at the equilibrium potential. Reproduced with permission.^[^ ^58^ ^]^ Copyright 2024, Wiley‐VCH. i) The decomposition energy barriers of Li_2_S adsorbed on CoS and Ni_0.5_Co_2.5_S_4_. Reproduced with permission.^[^ ^58^ ^]^ Copyright 2024, Wiley‐VCH.

Primarily, atom doping represents a universal method for the modification of sulfides. In the process of synthesis, hetero‐atoms are incorporated into the lattice of sulfides. This incorporation not only modifies the atomic arrangement but also gives rise to defects, which enhances diverse properties of sulfides, encompassing adsorption, catalytic activity, and electrical conductivity.^[^ ^55^ ^]^ For instance, Dong et al.^[^ ^56^ ^]^ fabricated a 2D ultrathin layered Co‐doped vanadium tetra sulfide/reduced graphene oxide (rGO) composite (x%Co‐VS_4_/rGO)—serving as the active sulfur host material in Li–S cells—through a facile hydrothermal approach (**Figure** **3**a). In this composite, the regular arrangement of the local lattice within VS_4_ was perturbed by the doped cobalt atoms. The perturbation engendered an increased number of defects, which functioned as active sites, thereby augmented the electrocatalytic activity (Figure 3b). DFT calculations showed that the adsorption energies of the 3%Co‐VS_4_/rGO catalyst exhibited spin polarization, and its adsorption energies with Li_2_S_4_, Li_2_S_6_, and Li_2_S_8_ were 1.76, 1.61, and 1.56 eV respectively (Figure 3c), which were higher than those of pure VS_4_. In addition, in situ X‐ray diffraction demonstrated that 3%Co‐VS_4_/rGO could promote the crystal‐phase transformation of α‐S_8_ to β‐S_8_ before and after charge–discharge, thus facilitating the redox reactions of lithium polysulfides (LiPSs) and ensures stability during cycling. At a high current density of 3 C, the S@3%Co‐VS_4_/rGO battery achieved a first discharge specific capacity of 633.1 mAh g^−1^, and it could stably cycle for 1000 times without a cliff‐like decline in capacity.

Drawing inspiration from the burr‐like structures in plant root systems, Dai et al.^[^ ^57^ ^]^ ingeniously designed an analogous architecture and utilized carbon nanotubes and flexible carbon nanofibers as starting materials. This designed structure was then modified with cobalt doped nickel sulfide nanoparticles, culminating in the formation of a composite material (Co‐NiS_2_@CNF‐CNT), which exhibits remarkable catalytic and adsorption properties. As vividly depicted in Figure 3d,e, the carbon nanofibers, which bear a resemblance to rhizomes, are intricately entwined by carbon nanotubes that are reminiscent of root hairs. This unique configuration gives rise to a highly efficient electron transport pathway. As a result, charge transfer is significantly accelerated, and the overall internal resistance is substantially reduced. Furthermore, DFT calculations reveal that electron‐deficient regions are generated in the vicinity of the Co element within NiS_2_. These regions not only enhance the interaction between the composite material and LiPSs but also enable Co‐NiS_2_ to possess a considerably stronger adsorption energy for various lithium polysulfides when compared to that of pure NiS_2_. The battery employing the novel Co‐NiS_2_@CNF‐CN material exhibited an impressively high specific discharge capacity of 951.4 mAh g^−1^ during the initial charge–discharge cycle under a large current density of 3 C. Remarkably, even at a rate of 5 C, the battery could operate normally for up to 3000 cycles (Figure 3f), highlighting its outstanding stability and discharge specific capacity. Subsequently, Wang et al.^[^ ^58^ ^]^ employed a hydrothermal method to oxidize in situ dissolved Ni foam and Co^2+^. Through this process, Ni‐doped Ni_x_Co_3‐x_S_4_ catalysts were successfully formed at high temperatures. Compared with monometallic spinel Co_3_S_4_, the introduction of Ni atoms reduced the crystallinity of the Co_3_S_4_ phase, which concurrently introduced a greater number of active sites. As a result, various polysulfides anchored on the catalyst surface were driven toward an optimal configurational state (Figure 3g). This remarkable change significantly decreased the energy barrier of the sulfur redox reaction (Figure 3h,i). Ultimately, the LSBs with the S@Ni_0.5_Co_2.5_S_4_/CNT cathode delivered a high initial specific capacity of 1189 mAh g^−1^ at a high rate of 5 C. Moreover, they still demonstrated excellent electrochemical performance even at low temperatures.

An optimal vacancy concentration can not only provide abundant active sites to maintain the balance between adsorptive and catalytic capacities, but also promote the phase and structure of TMSs to transform into a more stable state, thereby continuously driving the conversion of polysulfides.^[^ ^59^ ^]^ Owing to the fact that the formation energy of sulfur vacancies in Co‐MoS_2_ is lower than that in MoS_2_, this difference makes it easier to stabilize the 1T phase and sulfur vacancies.^[^ ^60^ ^]^ Thus, Co was doped into MoS_2_, which induced a phase transformation of 2H MoS_2_ into the 1T phase, accompanied by the formation of sulfur‐containing vacancies.^[^ ^61^ ^]^ These vacancies played a crucial role. They served as electron donors, endowing sulfur atoms with additional electrons. This electron‐rich environment facilitated electron transfer during redox reactions, which was a key factor in enhancing the overall electrochemical performance. Moreover, Shi et al.^[^ ^62^ ^]^ prepared a nanoflower‐shaped Fe^3+^/Fe^2+^@MoS_2_/S composite material as a sulfur coating matrix using a simple hydrothermal method. The mutual conversion of Fe^2+^and Fe^3+^ during redox reactions can induce dynamic structural changes in MoS_2_ catalysts. Not limited to this, the co‐doping strategy of Fe^2+^ and Fe^3+^ can also modulate the advantageous phase transition (2H→1T) of the MoS_2_ matrix, thereby further boosting the catalytic activity and effectively limiting the shuttle effect. Significantly, Fe^2+^ and Fe^3+^ directly participate in the redox process, with the change of valence and the concomitant migration of lithium‐ions. This process endows the Fe^3+^/Fe^2+^@MoS_2_/S cathode with additional specific capacity. As a result, it exhibits a remarkably high specific discharge capacity of 1900 mAh g^−1^ at a low current density of 0.1 C. Moreover, during 500 consecutive charge–discharge cycles conducted at a rate of 0.5 C, the cathode demonstrates an outstanding cycling stability, with a low‐capacity decay rate of merely 0.13% per cycle.

#### 2.2.2 Modulates Electron Orbitals

**Figure 4:**
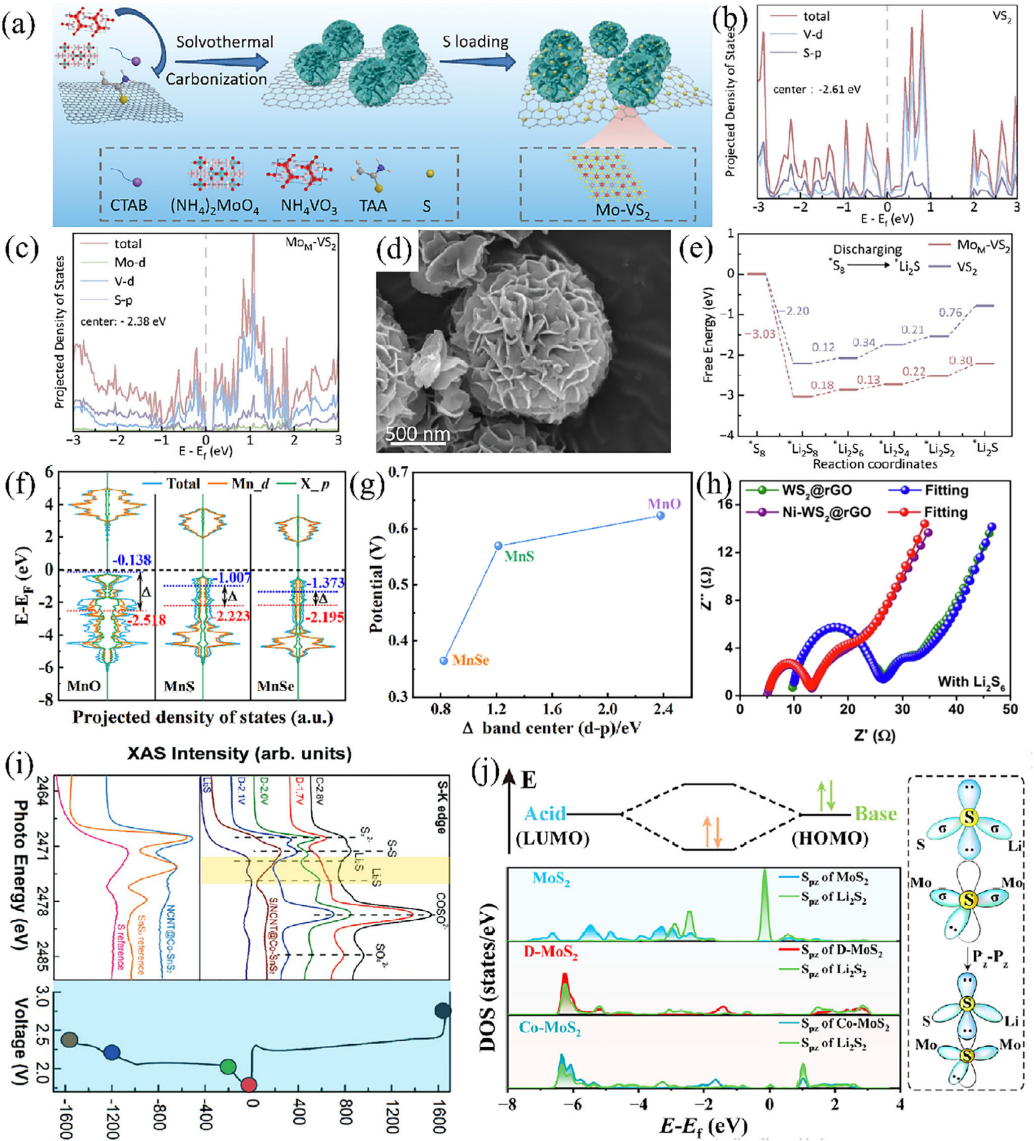
a) Schematic of the fabrication process of Mo‐VS_2_/rGO/S. Reproduced with permission.^[^ ^65^ ^]^ Copyright 2024, Wiley‐VCH. The comparison of Partial density of states (PDOS) for b) VS_2_ and c)Mo_M_‐VS_2_. Reproduced with permission.^[^ ^65^ ^]^ Copyright 2024, Wiley‐VCH. d) SEM images of Mo‐VS_2_/rGO. Reproduced with permission.^[^ ^65^ ^]^ Copyright 2024, Wiley‐VCH. e) Relative Gibbs free energy profiles and optimized adsorption models of sulfur species on the surface of VS_2_ and Mo‐VS_2_. Reproduced with permission.^[^ ^65^ ^]^ Copyright 2024, Wiley‐VCH. f) Density of states of the p bands of X and the d band of Mn in MnX. Reproduced with permission.^[^ ^66^ ^]^ Copyright 2022, Elsevier. g) Relationship between the Δ band center (d‐p) and Li–S redox potentials for MnX. Reproduced with permission.^[^ ^66^ ^]^ Copyright 2022, Elsevier. h) The corresponding EIS of the symmetric cells. Reproduced with permission.^[^ ^67^ ^]^ Copyright 2022, Elsevier. i) S K‐edge XANES of and S/CP@NCNT@Co‐SnS_2_ electrodes at different discharge/charge states. Reproduced with permission.^[^ ^68^ ^]^ Copyright 2019, Wiley‐VCH. j) DOS of S (from S, S_2_ , and d‐S_2_) and S (from Li_2_S_2_) onto the p_z_ orbit for MoS_2_ adsorbs Li_2_S_2_ , and a schematic for Lewis acid‐base interaction and the head‐on overlap between S (from d‐S_2_ or S_2_) and S (from Li_2_S). Reproduced with permission.^[^ ^69^ ^]^ Copyright 2025, Elsevier.

Studies have widely demonstrated that enhancing catalyst functionality by modulating d‐p orbital interactions to achieve high‐rate and long‐cycle performance in LSBs is effective, as exemplified by the single‐atom P and S co‐coordinated asymmetric configuration.^[^ ^63^ ^]^ Doping atoms are also capable of regulating the catalytic and adsorption properties by altering the d‐orbital centers of metal atoms in sulfides, which accelerates the conversion of polysulfides and improves the performance of LSBs.^[^ ^64^ ^]^ For example, considering the combined effect of base plane reactivity and intrinsic metallic properties, Xia et al.^[^ ^65^ ^]^ selected VS_2_ as the substrate to carry elemental sulfur. Employing a one‐step hydrothermal approach and subsequent post‐annealing treatment, Mo‐doped VS_2_ flower‐shaped microspheres (Mo‐VS_2_/rGO) were synthesized and grown on a reduced graphene oxide matrix (as depicted in **Figure** **4**a). Mo atoms, possessing properties analogous to those of V atoms, were chosen as the doping species for VS_2_. Mo exhibits an electron configuration distinct from that of V, enabling it to modulate the position of the d‐band center of V. Theoretical calculation results indicate that after doping with a certain concentration of Mo atoms, the d‐band center in VS_2_, originally located ≈−2.61 eV, shifts to −2.38 eV (as shown in Figure 4b,c). The upward shift of the orbital center significantly enhances the adsorption and catalytic capabilities of the VS_2_ catalyst, which helps to reduce the reaction energy barrier and accelerate the kinetic rate of polysulfide reactions. In addition, the flower‐shaped morphology (Figure 4d), characterized by abundant pores, is capable of efficiently mitigating the volume expansion effect associated with sulfur and offering sufficient space for the redox reactions of sulfur to occur, thus significantly enhancing performance. During the exothermic conversion of S_8_ to Li_2_S_8_, the reduction process on Mo‐VS_2_ proceeds more spontaneously than on pristine VS_2_. Mo‐VS_2_ shows a lower free energy of −3.03 eV as opposed to −2.2 eV for VS_2_, evidencing a stronger inclination toward spontaneity (Figure 4e). As a result, in the electrochemical performance test, the Mo‐VS_2_/rGO/S cathode exhibits a high initial specific discharge capacity of ≈1200 mAh g^−1^ at 0.2 C. Overall, the Mo‐VS_2_/rGO/S positive electrode exhibits significantly improved cycling stability and rate performance. Furthermore, anions exert distinct tuning effects on d‐p orbital centers, while variations in p‐band positions lead to differing catalytic promotion of polysulfide conversion processes. Liu et al.^[^ ^66^ ^]^ calculated the density of states (DOS) distributions of anionic p‐bands and Mn d‐bands in three catalysts (MnO, MnS, and MnSe) using DFT calculations. As shown in Figure 4f, owing to oxygen anions with higher electronegativity, the d‐orbital center of Mn cations in MnO lies closer to the Fermi level; this orbital distribution enables more electron donation, thereby endowing MnO with stronger adsorptive ability toward LiPSs. Additionally, due to the combined influence of atomic radius and electronegativity, the p‐band centers of non‐metallic anions exhibit a regular trend. Specifically, the Se p‐band center in MnSe undergoes a significant shift toward the Fermi level; this upward shift facilitates increased valence band electron density and boosts electron energy, thus promoting electron exchange in the Li_2_S_6_/Li_2_S reaction. Meanwhile, the energy gap between the d‐ and p‐band centers correlates positively with the Li_2_S_6_/Li_2_S reaction potential (Figure 4g), implying that MnSe endows LSBs with lower response overpotential, shorter response time, and larger response current during redox reactions. In summary, doping TMS catalysts with appropriate anions can result in materials with optimal band gap widths, while significantly modulating interfacial charge transfer behavior—thereby enhancing lithium‐sulfur electrochemical kinetics.

Through doping modification, improvements can be achieved in its resistance to self‐discharge, ionic conductivity, and the transport of lithium ions, such as Ni doped WS_2_.^[^ ^67^ ^]^ In this work, the layered structure of WS_2_ connected together by van der Waals forces exhibits convenient channels for lithium‐ion migration, and the doping of Ni atoms further enhances the polarity of WS_2_, thus adding more reaction sites to promote the conversion of polysulfides. From the alternating current impedance spectroscopy in Figure 4h, it can be seen that the Ni doping strategy has promoted the transfer rates of electrons and ions, and significantly reduced the overall internal resistance of the battery. Ultimately, even under high sulfur loading conditions, this battery was able to deliver a discharge specific capacity of 1160.8 mAh g^−1^ at 0.2 C, while maintaining stable cycling for 500 cycles at a high rate of 1 C. In addition, ion doping can promote the reaction kinetics of discharge and charge processes. Gao et al.^[^ ^68^ ^]^ ingeniously devised a Co‐doped SnS_2_ composite material that was anchored on N‐doped carbon nanotubes (NCNTs), denoted as Co‐SnS_2_@NCNT. Serving as a sulfur host cathode, it effectively promotes the conversion of LiPSs to Li_2_S and the reversible oxidation of Li_2_S. This phenomenon has been comprehensively verified through non‐in situ X‐ray absorption near‐edge structure (XANES) analysis. In the S K‐edge XANES of the S/Co‐SnS_2_@NCNT electrode, the S–S bonding feature at the energy level of 2471.6 eV (Figure 4i) vanishes during the reduction reaction process of sulfur from 2.0 to 1.7 V and reappears during the oxidation process of sulfur, which indicates that in the battery system with the S/Co‐SnS_2_@NCNT electrode, sulfur is completely reduced to Li_2_S and then oxidized back to elemental sulfur during the charging process. This reversible reaction greatly reduces the loss of active material before and after the charge–discharge cycles, while improving the efficiency of Li–S batteries. Therefore, the S/Co‐SnS_2_@NCNT electrode with a sulfur loading of 3 mg cm^−2^ presented a high initial discharge specific capacity of 1337.1 mAh g^−1^ and retained a high reversible specific capacity of 1004.3 mAh g^−1^ even after 100 cycles at a current density of 1.3 mA cm^−2^. The introduction of dopant atoms also generates a certain concentration of vacancies, which not only activates inert basal planes and increases the number of active sites, but also enables precise tuning of interactions between the atomic orbitals of catalysts and polysulfides by regulating the ratio of vacancies to dopant atoms. Jiang et al.^[^ ^69^ ^]^ proposed a modification strategy where Co atoms and Mo vacancies are co‐anchored on the MoS_2_ basal plane, enabling p‐p orbital covalent coupling on the MoS_2_ surface. This precisely tunes the orbital orientation of surface sulfur atoms in MoS_2_, thereby fine‐regulating their interactions with lithium and sulfur sites in polysulfide species, and ultimately achieving efficient liquid‐solid conversion. Combining theoretical modeling and experimental validation, it has been confirmed that there is strong coupling between the p orbitals of unsaturated surface sulfur atoms and the p orbitals of short‐chain polysulfides. In d‐MoS_2_ and Co‐MoS_2_, the maximal face‐on overlap between the p_z_ orbitals of coordinatively unsaturated S and the p_z_ orbitals of S atoms in Li_2_S_2_ indicates the formation of robust S–S bonds (Figure 4j), thus accelerating the conversion kinetics from Li_2_S_4_ to Li_2_S_2_/Li_2_S. Ultimately, batteries incorporating Co‐MoS_2_@NC@CNT retain long‐term cycling stability even at a 2 C rate.

#### 2.2.3 Different Effects of Heteroatom Types and Doping Sites

**Figure 5:**
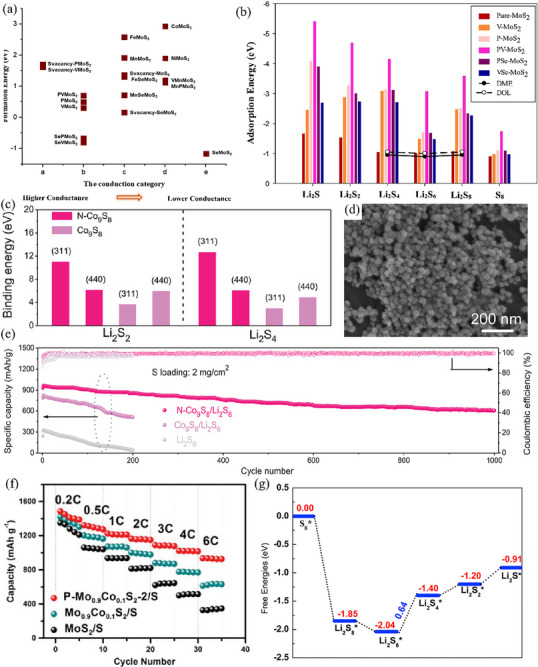
a) Formation energies of doped‐MoS_2_ and conductivity. Reproduced with permission.^[^ ^72^ ^]^ Copyright 2024, Elsevier. b) Adsorption energies of polysulfides on MoS_2_ doped with various atoms and the adsorption energies of polysulfides with electrolyte solvent molecules (DME and DOL). Reproduced with permission.^[^ ^72^ ^]^ Copyright 2024, Elsevier. c) SEM image for the N‐Co_9_S_8_ nanoparticles. Reproduced with permission.^[^ ^73^ ^]^ Copyright 2020, Wiley‐VCH. d) DFT calculation shows a significant increase in binding energies of N‐Co_9_S_8_ nanoparticles in the Li_2_S_6_ solution. Reproduced with permission.^[^ ^73^ ^]^ Copyright 2020, Wiley‐VCH. e) Cycling stability of the N‐Co_9_S_8_/Li_2_S_6_ cathodes for 1000 cycles. Reproduced with permission.^[^ ^73^ ^]^ Copyright 2020, Wiley‐VCH. f) Rate performance comparison at different C‐rates. Reproduced with permission.^[^ ^76^ ^]^ Copyright 2019, Wiley‐VCH. g) Energy profiles for the reduction of S_8_ on CoS substrate. Reproduced with permission.^[^ ^79^ ^]^ Copyright 2021, Elsevier.

In order to investigate the influence of different doped atoms on the catalytic effect, Zhao et al.^[^ ^70^ ^]^ introduced a diverse range of transition metal elements, including Ni, Fe, Co, Ti, Cu, V, and Mn, as dopants into SnSe. The Gibbs free energy results demonstrate that although all doping treatments notably enhance the adsorption capacity of LiPSs and S_8_, not all doping methods can lead to a reduction in the length of the Sn‐S bonds formed between SnSe and polysulfides. This finding indicates that different doping atoms will exert different catalytic mechanisms in the same matrix, resulting in varied enhancements on improving the performance of LSBs. Building upon this foundation, analogous studies have been conducted to investigate the properties of sulfides. Zhang incorporated various metal ions (Mn^2+^, Fe^2+^, Co^2+^, Ni^2+^, Cu^2+^) into zinc sulfide (ZnS) and examined their impacts on the catalytic activity and adsorption capacity of the substrate.^[^ ^71^ ^]^ According to the disparities in the peak current of the cyclic voltammetry (CV) curves of symmetrical batteries, the catalytic ability of ZnS doped with metal ions ranging from Mn to Cu exhibits a tendency of initially increasing and then decreasing. This phenomenon is attributed to the fact that the enhanced adsorption capacity leads to the deposition of a layer of solid Li_2_S on the catalyst surface, resulting in surface passivation and a decrease in catalytic activity. It is evident that by changing the type of doping atoms, the relationship between catalysis and adsorption can be adjusted, thereby obtaining more excellent sulfide catalysts. Similarly, Abbasi et al.^[^ ^72^ ^]^ focused on studying the modification effects of different dopants on layered MoS_2_ catalysts through first principles calculations, including metal dopants (V, Ni, Co, Mn, and Fe), non‐metal dopants (Se and P), and co‐doping situation. The final findings reveal that, in comparison with other dopants, phosphorus (P) and vanadium (V) dopants in the molybdenum disulfide (MoS_2_) structure display lower formation energies for lithium sulfide (Li_2_S), while also significantly improving conductivity (**Figure** **5**a). In addition, they demonstrate superior adsorption energy required for effectively immobilizing LiPSs. It is worth noting that the synergistic effect observed in PV‐MoS_2_ samples co‐doped with phosphorus and vanadium significantly enhances the binding energy of Li_2_S_n_ species on MoS_2_ monolayers (Figure 5b), which is much higher than the adsorption energy of MoS_2_ doped with single elements.

Moreover, anionic dopants have been proven to strengthen the chemical interaction between sulfide catalysts and LiPSs. Liu et al. synthesized spherical N‐doped Co_9_S_8_ nanoparticles (Figure 5c) and demonstrated the nitrogen doping strategy could concurrently enhance the immobilization of LiPSs and the redox catalytic activity of Co_9_S_8_ nanoparticles in Li–S batteries.^[^ ^73^ ^]^ This is because, on the one hand, N doping can reduce the intrinsic activation potential barrier during the catalysis, which is an effective method for optimizing the electrochemical performance of Li–S batteries. On the other hand, Li‐N bonds are more advantageous than Li–S bonds for anchoring LiPSs within the sulfur host cathode. Density functional theory calculations further demonstrated that nitrogen doping enhanced the bonding between LiPSs and the surface of Co_9_S_8_ nanoparticles (Figure 5d). With respect to cycling performance, the cathode with N‐Co_9_S_8_ nanoparticles exhibited an extremely low‐capacity decay rate of only 0.037% per cycle after 1000 cycles at a current density of 1 A g^−1^ (Figure 5e). These results highlight that doping with non‐metallic elements can effectively improve the performance of LSBs.

Subsequently, aiming to regulate the orbital states of metal and non‐metal atoms, phosphorus (P), which features a relatively low electronegativity,^[^ ^74^ ^]^ was incorporated into MoS_2_ through doping.^[^ ^75^ ^]^ Experimental findings indicated that the orbitals of P underwent hybridization with the 2p orbitals of S and the 3d orbitals of Mo. This hybridization process not only significantly enhanced the electron transfer capability but also led to the formation of stronger chemical bonds between the catalyst and polysulfides. As a result, the interaction between MoS_2_ and LiPSs/Li_2_S was substantially strengthened, effectively curbing the shuttle effect of polysulfides. Building on this, Lee and his research team engineered a MoS_2_ material co‐doped with both Co and P, which was applied as the cathode material for LSBs.^[^ ^76^ ^]^ In this design, co‐dopants display significant synergistic effects. Specifically, the incorporation of Co dopants increases electron transport pathways within the cathode, thereby promoting improved electrical conductivity of the MoS_2_ matrix and ensuring adequate and more reversible utilization of active materials. Additionally, the incorporation of non‐metallic P dopants forms extra Mo‐P and Co‐P coordination sites on the matrix surface. This enhances the catalytic activity of MoS_2_ for polysulfides, ultimately accelerating the reaction kinetics of sulfur in the cathode and endowing it with the capacity to endure high‐current charge–discharge processes. As a result, the battery showcased outstanding high‐rate performance (Figure 5f). Wei and his colleagues designed a FeS_2_‐based material co‐doped with both metal elements Co, Ni and non‐metal elements P, O, N, and Se. DFT calculations showed that compared to other co‐doped varieties of FeS_2_, the FeS_2_ co‐doped with Co and either P or O exhibits a more pronounced lattice distortion effect and lower formation energy.^[^ ^77^ ^]^ This characteristic is more favorable for the migration of lithium ions and the conduction of electrons. In a similar vein, doping cobalt disulfide (CoS_2_) with phosphorus (P) also leads to an enhancement in its catalytic activity.^[^ ^74^ ^]^ Last but not least, apart from the type of doping ions, the doping position and content also emerge as crucial factors influencing the doping effect.^[^ ^78^ ^]^ Taking Co‐doped MoS_2_ as an illustrative example,^[^ ^79^ ^]^ elements such as Mn, Fe, Co, Ni, etc. are respectively doped at the metal sites (Mo) and non‐metal sites (S) of MoS_2_. Doping MoS_2_ at the Mo site does not significantly improve the bonding strength with LiPSs. On the contrary, the binding energy between MoS_2_ and polysulfides is increased by doping at the S site, which is attributed to the stronger orbital overlap between the electron orbitals of Co and the orbitals of S within Li_2_S_4_. In addition, MoS_2_ doped by substituting S atoms shows good catalytic activity for the conversion of LiPSs at a low Gibbs free energy. The MoS_2_ doped by replacing S sites can reach a lower Gibbs free energy level in the redox reaction and display excellent catalytic activity for the decomposition of Li_2_S (Figure 5g).

In summary,^[^ ^80^, ^81^, ^82^, ^83^ ^]^ when compared with undoped TMSs, heteroatom doping presents numerous advantages. It not only boosts the conductivity of TMS catalysts, provides more chemical anchoring sites for LiPSs, but also accelerates charge transfer between polysulfides in the electrode and the electrolyte. Consequently, the catalytic activity is enhanced, the sulfur conversion rate is improved, and the overall electrochemical performance is optimized. Significantly, it must be emphasized that doping can simultaneously bring about changes in the lattice distortion, spin polarization, d‐band center, and the generation of vacancy defects. Thus, controlling the electronic structure through doping is a complex endeavor that requires comprehensive exploration of these factors. Moreover, the development of directional doping at specific sites should be promoted to achieve higher catalytic activity. As the content of the doping element rises, lattice distortion will be aggravated, thereby reducing the structural stability of sulfides. And, it is of crucial importance to strike a balance between the doping atoms and the sulfide matrix and to ensure stable cathode host materials for the realization of high‐efficiency Li–S cells.

### 2.3 Multiple‐Ion TMSs

#### 2.3.1 Bimetallic TMSs

Based on the aforementioned research, the incorporation of heteroatoms not only augments the adsorption and catalytic sites for polysulfides but also enables the manifestation of synergistic effects among different components, which deservedly accelerates the conversion of LiPSs.^[^ ^83^ ^]^ Consequently, sulfides composed of multiple metal ions have also been developed and implemented in LSBs.^[^ ^84^, ^85^, ^86^ ^]^ Analogous to heteroatom‐doped sulfides, multi‐metallic TMSs, which contain two or more metal elements, possess an abundance of chemical sites with a strong adsorption capacity and catalytic ability. In contrast to doped single‐metal sulfides, the microstructures of which are liable to be damaged as the content of impurity atoms rises, multi‐metal transition metal sulfides possess a distinctive and stable crystal structure.^[^ ^87^ ^]^ This characteristic effectively prevents the positive electrode from collapsing and detaching during extended cycling, thereby guaranteeing the high‐performance operation of Li–S batteries.

**Figure 6:**
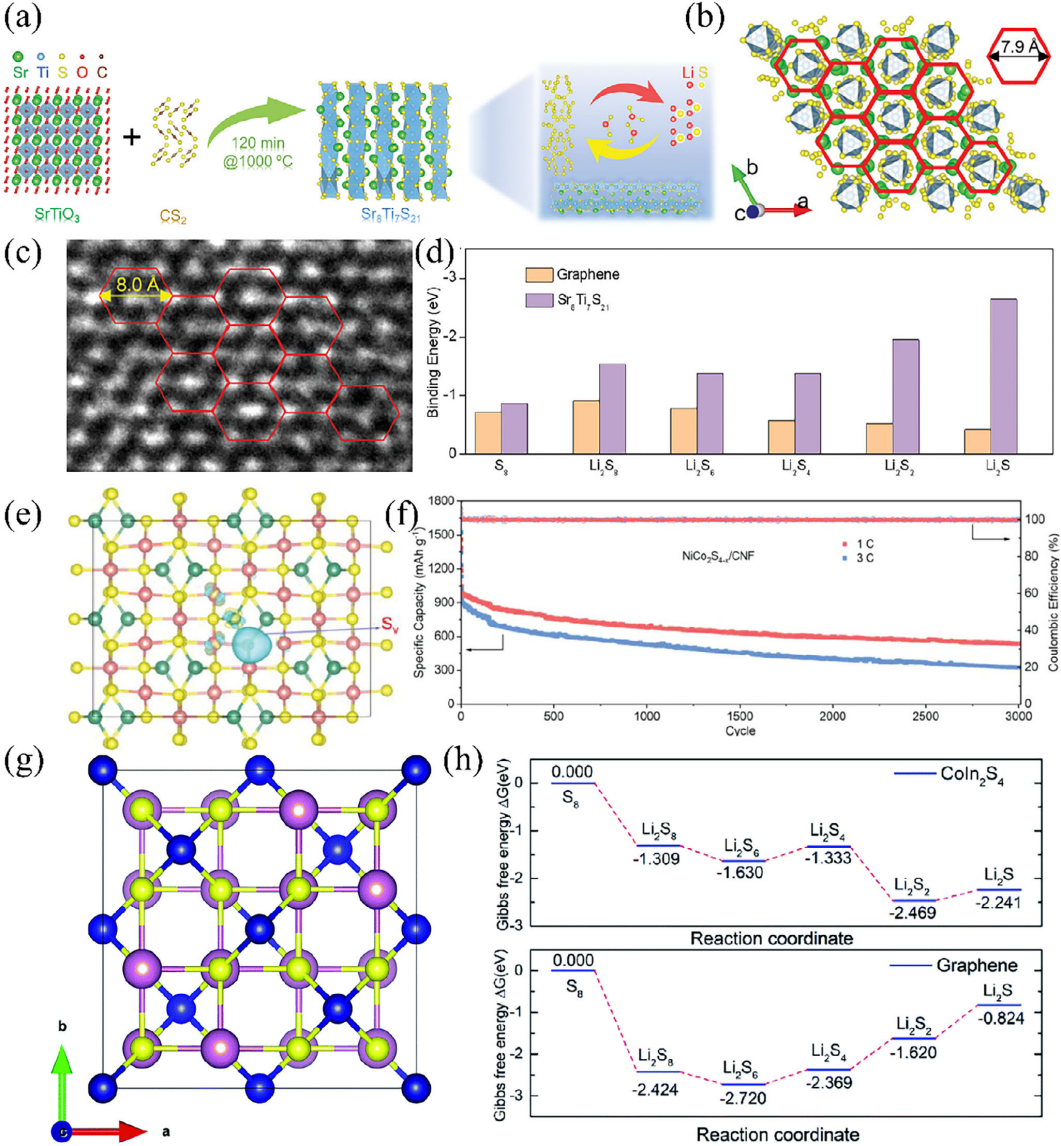
a) Schematic diagram of the synthesis process used to produce the Sr_8_Ti_7_S_21_ powder. Reproduced with permission.^[^ ^91^ ^]^ Copyright 2024, Wiley‐VCH. b) Crystal structure of Sr_8_Ti_7_S_21_ from the c axis. Reproduced with permission.^[^ ^91^ ^]^ Copyright 2024, Wiley‐VCH. c) HRTEM image of a Sr_8_Ti_7_S_21_ powder with hexagons formed by Sr noted in red. Reproduced with permission.^[^ ^91^ ^]^ Copyright 2024, Wiley‐VCH. d) Binding energies between LiPSs and graphene or Sr_8_Ti_7_S_21_ surfaces. Reproduced with permission.^[^ ^91^ ^]^ Copyright 2024, Wiley‐VCH. e) Electronic deficient region resulted from sulfur vacancy. Reproduced with permission.^[^ ^48^ ^]^ Copyright 2023, Wiley‐VCH. f) Ultra‐long life tests of Li–S batteries with NiCo_2_S_4‐x_/CNF interlayer at 1 C and 3 C for 3000 cycles. Reproduced with permission.^[^ ^48^ ^]^ Copyright 2023, Wiley‐VCH. g) Crystal structure of CIS. The blue, pink, and yellow balls represent Co, In, and S, respectively. Reproduced with permission.^[^ ^93^ ^]^ Copyright 2021, Royal Society of Chemistry. h) Energy profiles of different sulfur species on the surface of CIS or graphene. Reproduced with permission.^[^ ^93^ ^]^ Copyright 2021, Royal Society of Chemistry.

Bimetallic TMSs were first studied and applied. For example, inspired by the fact that chalcogenide perovskites usually possess excellent charge transport ability,^[^ ^88^ ^]^ high light absorption ability,^[^ ^89^ ^]^ and numerous chemical sites,^[^ ^90^ ^]^ Yang et al.^[^ ^91^ ^]^ proposed using perovskite‐type materials with abundant sites as sulfur hosts, and then synthesized quasi‐1D Sr_8_Ti_7_S_21_ by high‐temperature sulfurization of SrTiO_3_ powder (**Figure** **6**a). The powdered material belongs to the chalcogenide perovskite structure, where the Ti‐S_6_ octahedrons are connected to each other through a shared triangular crystal plane and are separated by Sr ions (Figure 6b), forming a 1D chain structure that provides a convenient electron and lithium‐ion transport channel. This point is also confirmed by the high‐resolution transmission electron microscopy (HRTEM) images (Figure 6c). The distance between two opposite fixed points is ≈8 Å, which is similar to the crystal structure parameters of chalcogenide perovskites. Furthermore, Sr_8_Ti_7_S_21_ demonstrates the potential to form Li–S and double S‐Sr/Ti bonds, which endow the material with the ability to interact robustly with lithium polysulfides through multi‐bond effects. Density functional theory calculations reveal that the binding energy of Sr_8_Ti_7_S_21_ with LiPSs is higher than that of graphene (Figure 6d). Notably, the difference in the adsorption energy is more pronounced for Li_2_S, which indicates that Sr_8_Ti_7_S_21_ will display dual catalytic capabilities during the redox reaction process of the battery. Consequently, the electrochemical performance of the battery will be enhanced. At a rate of 0.2 C, the S@Sr_8_Ti_7_S_21_ cathode exhibited an initial discharge specific capacity of 1266 mAh g^−1^ and maintained a reversible specific capacity of 1198 mAh g^−1^ after 100 cycles, with an overall capacity loss of only 0.054%. After 400 cycles of testing at a high rate of 1 C, the reversible specific capacity of the S@Sr_8_Ti_7_S_21_ electrode was still maintained at 520 mAh g^−1^, with an average capacity decay of only 0.078% per cycle, demonstrating excellent cycling stability. At the same time, these results also demonstrate that Sr_8_Ti_7_S_21_ powder material has a stable structure and is not easily damaged at high current densities. Furthermore, Dai et al.^[^ ^48^ ^]^ synthesized NiCo_2_S_4_ nanosheets on carbon nanofiber (CNF) membranes using the hydrothermal method, S deficiencies were induced in the lattice of the nanosheets through thermal reduction (as shown in the blue area in Figure 6e), ultimately preparing NiCo_2_S_4‐x_/CNF composite materials containing S vacancy defects. This defect can cause atomic rearrangement around vacancies, forming locally unstable regions, thereby changing the electronic density of states of the material and becoming active sites that enhance catalytic ability. DFT calculations show that NiCo_2_S_4‐x_ has stronger adsorption energy and higher electronic density of states for LiPSs, indicating that NiCo_2_S_4‐x_ will promote the conversion of polysulfides faster. Therefore, the initial discharge specific capacity of NiCo_2_S_4‐x_/CNF battery can reach 1291.9 mAh g^−1^ at a charge discharge rate of 0.2 C, and it can still exhibit a discharge specific capacity of 922.8 mAh g^−1^ after 500 cycles, with a decay rate of only 0.057% per cycle, much lower than the cycling decay rates of NiCo_2_S_4_/CNF (0.069%) and CNF (0.079%). At the same time, after 3000 constant current charge–discharge cycles under high‐rate tests at 1 C and 3 C, the reversible capacities of 535.9and 326.8 mAh g^−1^ were still maintained, demonstrating excellent cycling performance (Figure 6f).

Leveraging its inherent strong polarity, the binary metal sulfide, specifically CoIn_2_S_4_, has found extensive applications in the realm of photoelectron catalytic hydrogen production.^[^ ^92^ ^]^ Inspired by the insights, Hu et al. employed the solvothermal method to synthesize surface‐porous CoIn_2_S_4_ nanoparticles.^[^ ^93^ ^]^ Subsequently, heat treatment was carried out to further improve the crystallinity of the material. The crystal structure of CoIn_2_S_4_ consists of Co^2+^ ions and In^3+^ ions, which respectively occupy the octahedral and tetrahedral voids within the closed‐packed S‐anion lattice (Figure 6g). This structural feature imparts strong polarity to the material, endowing it with a remarkable adsorption capacity for polysulfides. Additionally, in situ Raman measurements and theoretical calculations have verified that the CoIn_2_S_4_ catalyst substantially accelerates the conversion rate of the rate‐determining step and lowers the energy barrier for the transformation from S to Li_2_S (Figure 6h). As a result, LSBs incorporating CoIn_2_S_4_ are capable of delivering a specific capacity of 686 mAh g^−1^ even at a high charge–discharge rate of 3 C. Moreover, they showcase outstanding electrochemical performance under high sulfur loading conditions.

**Figure 7:**
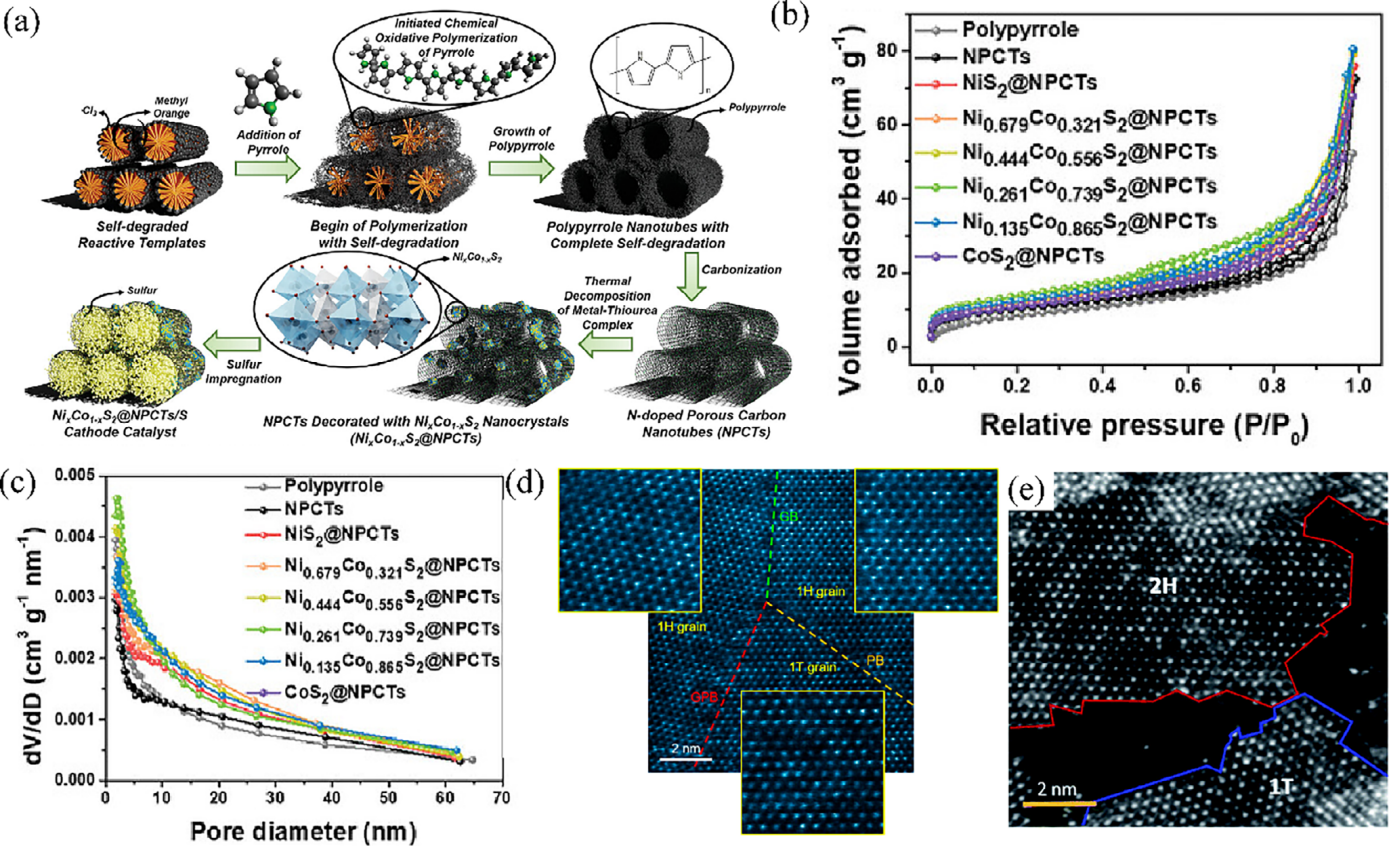
a) Schematic showing the overall synthesis process the Ni_x_Co_1‐x_S_2_@NPCTs composites. Reproduced with permission.^[^ ^94^ ^]^ Copyright 2024, Wiley‐VCH. b) N_2_ adsorption/desorption curves and c) pore size distributions of the samples, respectively. Reproduced with permission.^[^ ^94^ ^]^ Copyright 2024, Wiley‐VCH. d) Atom‐resolved HAADF−STEM image of the distribution of 1T and 1H phases. Reproduced with permission.^[^ ^97^ ^]^ Copyright 2018, American Chemical Society. e) HAADF‐STEM image for a monolayer section of Mo_0.5_W_0.5_S_2_. Reproduced with permission.^[^ ^98^ ^]^ Copyright 2020, Royal Society of Chemistry.

By adjusting the ratio of several metal atoms, the catalytic effect can also be controlled, thereby enabling bimetallic sulfide catalysts to exhibit better performance in batteries. For instance, Ji et al.^[^ ^94^ ^]^ explored the characteristics of Ni_x_Co_1‐x_S_2_ binary metal sulfides with different Ni and Co contents, where x and 1‐x correspond to the actual atomic concentration ratios of Ni and Co determined by inductively coupled plasma atomic emission spectroscopy (ICP‐AES). Six types of Ni_x_Co_1‐x_S_2_ samples were obtained through the synthesis method shown in **Figure** **7**a, and were simultaneously combined with nitrogen doped porous carbon nanotubes (NPCTs) to enhance their sulfur loading capacity. The results implied that the catalyst groups were NiS_2_@NPCTs, CoS_2_@NPCTs, Ni_0.679_Co_0.321_S_2_@NPCTs, Ni_0.444_Co_0.556_S_2_@NPCTs, Ni_0.261_Co_0.739_S_2_@NPCTs, and Ni_0.135_Co_0.865_S_2_@NPCTs, which shared fundamental crystal structures that are layered morphologies composed of octahedral TM‐S (TM = Ni, Co) stacking, however, the addition of nickel and cobalt at different concentrations can alter certain properties of the material. The N_2_ adsorption‐desorption isotherms and pore size distribution curves in Figure 7b,c indicate that controlling the Ni/Co ratio leads to slight changes in Brunauer‐Emmett‐Teller (BET) texture parameters. Among these composites, Ni_0.261_Co_0.739_S_2_@NPCTs exhibits the highest specific surface area (48.5 m^2^ g^−1^), with pore sizes primarily distributed in the 5–20 nm range. This characteristic facilitates higher sulfur loading and the formation of structurally stable cathode materials. The difference in specific surface area will directly affect the sulfur loading capacity, thereby affecting the electrochemical performance. The alteration of binding energy triggered by the coexistence of Ni_Oh_^2+^and Co_Oh_^2+^sites on the Ni_Oh_^2+^‐S‐Co_Oh_^2+^ skeleton can synergistically convert LiPSs to Li_2_S_2_, and then activate diffusion controlled 3D Li_2_S nucleation through the relaxation of S–S bonds in Li_2_S_2_.^[^ ^95^ ^]^ The catalytic sites of Ni_0.261_Co_0.739_S_2_ can maintain the activation state of sustainable sulfur redox processes, which is quite hindered for two types of single metal sulfides. Therefore, Ni_0.261_Co_0.739_S_2_@NPCTs will demonstrate sustainable catalytic effects and excellent electrochemical performance. Among samples with different nickel‐cobalt ratios, Ni_0.261_Co_0.739_S_2_@NPCTs cell demonstrates a lower degree of polarization and superior rate performance. Specifically, at a current density of 1 C, the Ni_0.261_Co_0.739_S_2_@NPCTs‐based battery exhibits the optimal performance and the highest cycle stability. It has a retention rate of 72.8% and a low‐capacity decay of merely 0.054% per cycle, which indicates the excellent structural stability of the material.

Likewise, for Mo_x_W_y_S catalyst, the 1T phase structure has better electronic conductivity, making it more catalytically active, while the 2H phase is more favorable for Li^+^ diffusion. The mixed 1T + 2H phase state can give rise to more potent synergistic effects, thereby enabling the acquisition of highly efficient catalysts. Choi et al.^[^ ^96^ ^]^ developed a scalable synthesis method with compositional tunability in few layer Mo_1‐x_W_x_S_2_ compounds. Chen et al.^[^ ^97^ ^]^ achieved a systematic phase transition between 1T and 2H phases by changing the composition of W in MoS_2_ through chemical vapor deposition, thus obtaining Mo_1‐x_W_x_S_2_ nanolayered catalysts with coexisting phases (Figure 7d). In light of this concept, Bhoyate and his colleagues initially employed the co‐sputtering technique to deposit two metals, namely Mo and W, onto paper composed of carbon nanotubes (CNTs).^[^ ^98^ ^]^ Subsequently, this paper was transferred into a low‐pressure chemical vapor deposition chamber, where sulfidation was carried out at 600 °C. As a result, the Mo_0.5_W_0.5_S_2_ material with co‐existent metallic (1T) and semiconducting (2H) phases was successfully synthesized (Figure 7e). Electrochemical performance tests clearly indicated that this catalyst with the co‐presence of two phases significantly enhanced the migration rates of both electrons and lithium ions. It effectively promoted the kinetics of the redox reaction process and maximally curbed the shuttle effect. Furthermore, MoS_2_ with coexisting 2H and 1T phases exhibits superior structural stability relative to single‐phase counterparts, thereby ensuring the structural integrity of the cathode during cycling. The LSBs equipped with the Mo_0.5_W_0.5_S_2_‐CNTs‐S cathode manifested outstanding rate performance and cycling stability. Specifically, at a current density of 0.5 C, it could still retain a high reversible specific capacity of 790 mAh g^−1^ even after 400 stable cycles.

#### 2.3.2 Multimetallic Sulfides and High‐Entropy TMSs

**Figure 8:**
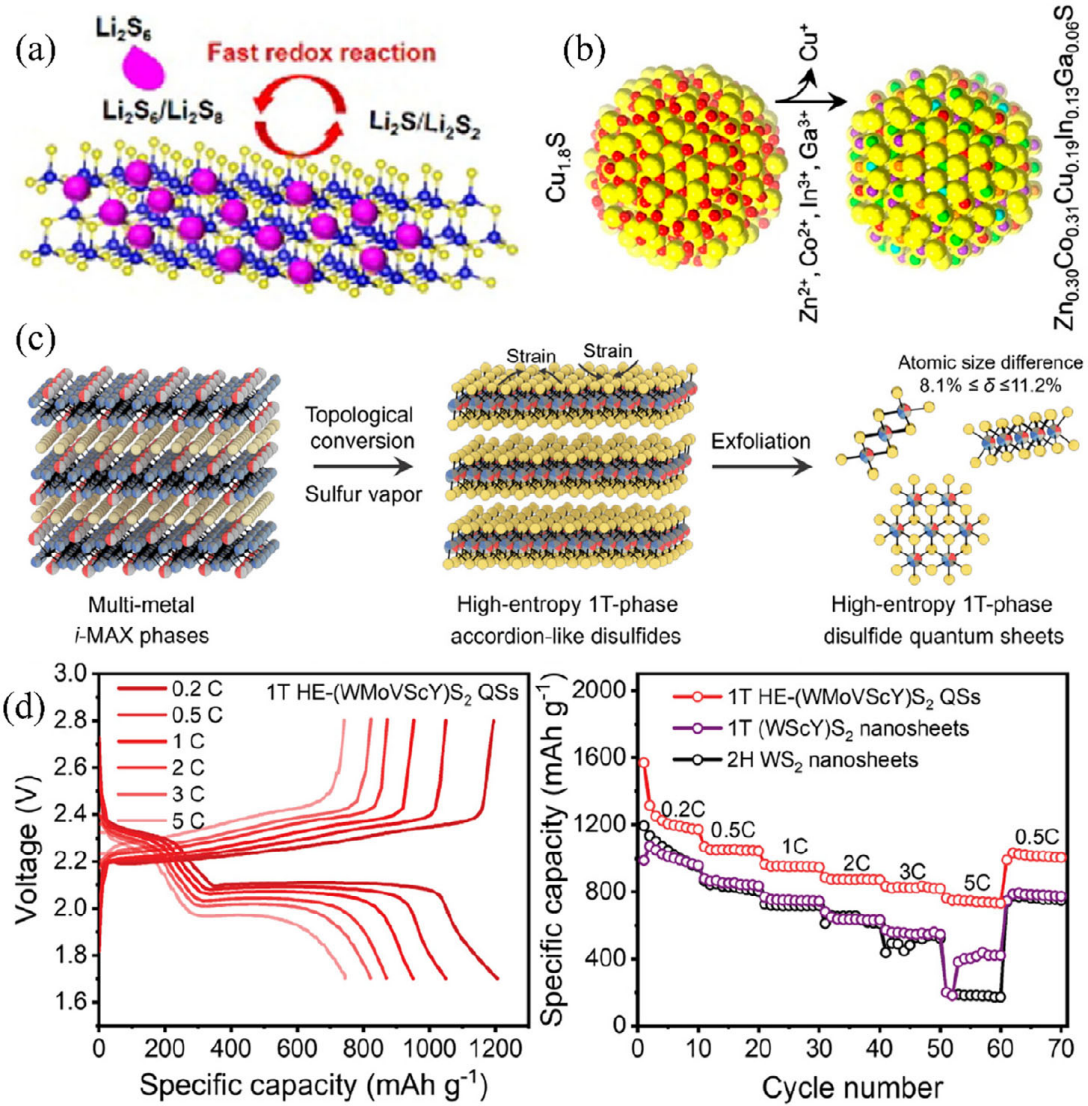
a) Schematic illustration of fast redox reaction and electron transfer processes. Reproduced with permission.^[^ ^99^ ^]^ Copyright 2021, Elsevier. b) Schematic showing the synthesis of Zn_0.30_Co_0.31_Cu_0.19_In_0.13_Ga_0.06_S nanoparticles. Reproduced with permission.^[^ ^101^ ^]^ Copyright 2023, American Chemical Society. c) Schematic illustration of high‐entropy 1T‐phase quantum sheets under sulfur vapor at high temperatures and subsequent exfoliation treatment. Reproduced with permission.^[^ ^102^ ^]^ Copyright 2025, Wiley‐VCH. d) Voltage profiles and rate performance at various rates from 0.2 C to 5 C. Reproduced with permission.^[^ ^102^ ^]^ Copyright 2025, Wiley‐VCH.

To further harness and maximize the potential of multiple metal ions, sulfides incorporating more than two metal species have been ingeniously designed and successfully implemented in LSBs. For example, Chen et al.^[^ ^99^ ^]^ employed a one‐step thermal decomposition synthesis approach to fabricate a Cu, Zn, Sn‐based 2D layered ternary metal sulfide catalyst. The profusion of catalytic sites contributed by Cu, Zn, and Sn atoms within the crystal lattice synergistically facilitated the conversion of diverse polysulfides, thereby markedly curbing the notorious shuttle effect (**Figure** **8**a). Additionally, the highly crystalline nanosheets effectively diminished the charge transfer resistance. These advantageous features comprehensively mitigated existing issues and substantially enhanced the overall performance. As a result, the Li–S cells attained an impressively high discharge specific capacity of 1200 mAh g^−1^ and maintained remarkable stability throughout 1000 cycles. In addition, taking into account the integration of diverse advantages inherent in different TMSs, as well as the synergistic interplay of multiple metals, high‐entropy materials, including high‐entropy transition metal compounds comprising over five metal elements, have been applied in LSBs. High‐entropy materials featuring a single‐phase crystal structure possess pronounced benefits of lattice distortion and the cocktail effect, exhibiting remarkable synergistic effects and unparalleled activity.^[^ ^100^ ^]^ For example, Abruna et al.^[^ ^101^ ^]^ incorporated Cu, Co, and Zn along with Ga and In into sulfide materials to balance charges and designed the high‐entropy sulfide Zn_0.30_Co_0.31_Cu_0.19_In_0.13_Ga_0.06_S to boost redox kinetics and mitigate the shuttle effect in LSBs (Figure 8b). It is worth noting that although CuGaS_2_ and CuInS_2_ catalyze the reduction reaction, they do not show the catalytic oxidation reaction ability of metal sulfides, while high‐entropy sulfides display remarkably enhanced oxidation kinetics owing to the synergistic interaction among components. The study further revealed that during extended charge–discharge cycles, copper in the high‐entropy sulfides was leached out in the form of ions, accompanied by lower crystallinity and smaller particles within the high entropy sulfides, which indicated that stabilizing the existing forms of various elements was a key factor for improving battery performance and an urgent problem to solve in high entropy materials.

To address the issue that the basal planes of transition‐metal dichalcogenides are prone to sliding when disturbed and to increase the content of the metallic 1T phase, Wang et al.^[^ ^102^ ^]^ employed a high‐entropy strategy and introduced multiple metal atoms (W, Mo, V, Sc, Y) with significant size differences into the dichalcogenides. Ultimately, they prepared 1T‐phase high‐entropy dichalcogenides through a combination of powder metallurgy and high‐temperature calcination (Figure 8c). Atoms of different sizes increased the lateral dimensions of the nanosheets, thereby delaying the slippage between crystal planes. However, owing to the significant differences in properties and poor compatibility among these transition metal atoms, the crystal structure is in a high‐strain state, which in turn induces the proneness of high‐index crystal planes to fracture and the formation of numerous high‐entropy disulfides with a quantum sheet morphology. During this process, edge sites with high catalytic activity are highly exposed, significantly enhancing the catalytic and adsorption abilities toward polysulfides. Simultaneously, the presence of the 1T phase improves the conductivity of this high‐entropy material, endowing it with low charge transfer resistance. Furthermore, numerous microscopic voids exist between the quantum sheets, which further enhance the wettability between the electrode and electrolyte, thereby facilitating fast lithium‐ion diffusion and in turn accelerating the redox reaction kinetics of sulfur. Additionally, the crystal structure of the quantum sheets formed post‐fracture exhibits high stability, ensuring its ability to maintain efficient and stable catalytic performance even under high current densities. Finally, the LSBs using this high‐entropy dichalcogenide demonstrated excellent rate performance. It even exhibited a specific discharge capacity of 744 mAh g^−1^ at a high current density of 5 C (Figure 8d). Moreover, the frequently employed metallic high‐entropy alloy FeCoNiMnZn^[^ ^103^ ^]^ and Mn_x_FeCoNiCu^[^ ^104^ ^]^ has been verified to be capable of enhancing the reaction kinetics during both the discharging and charging procedures of Li–S cells concurrently. Despite the outstanding performance of high‐entropy materials in LSBs, owing to their complex and diverse compositions, the reaction mechanisms within the battery remain elusive up to now.

Furthermore, there are no existing studies regarding the regulation of lattice distortion in high‐entropy materials and its correlation with catalytic activity. Considering that high‐entropy materials entail a vast and intricate array of elements, investigating their properties via experimental approaches not only demands substantial time and labor but also poses challenges in yielding precise outcomes. Thus, the utilization of specific high‐throughput computational methods to support experimental research can significantly boost efficiency. In addition, it is essential to design a wider range of high‐entropy materials, including high‐entropy oxides, high‐entropy fluorides, and high‐entropy chlorides.^[^ ^87^, ^105^ ^]^

### 2.4 Heterostructures of TMSs

#### 2.4.1 Increase the Number of Active Sites and Electronic Conductivity

**Figure 9:**
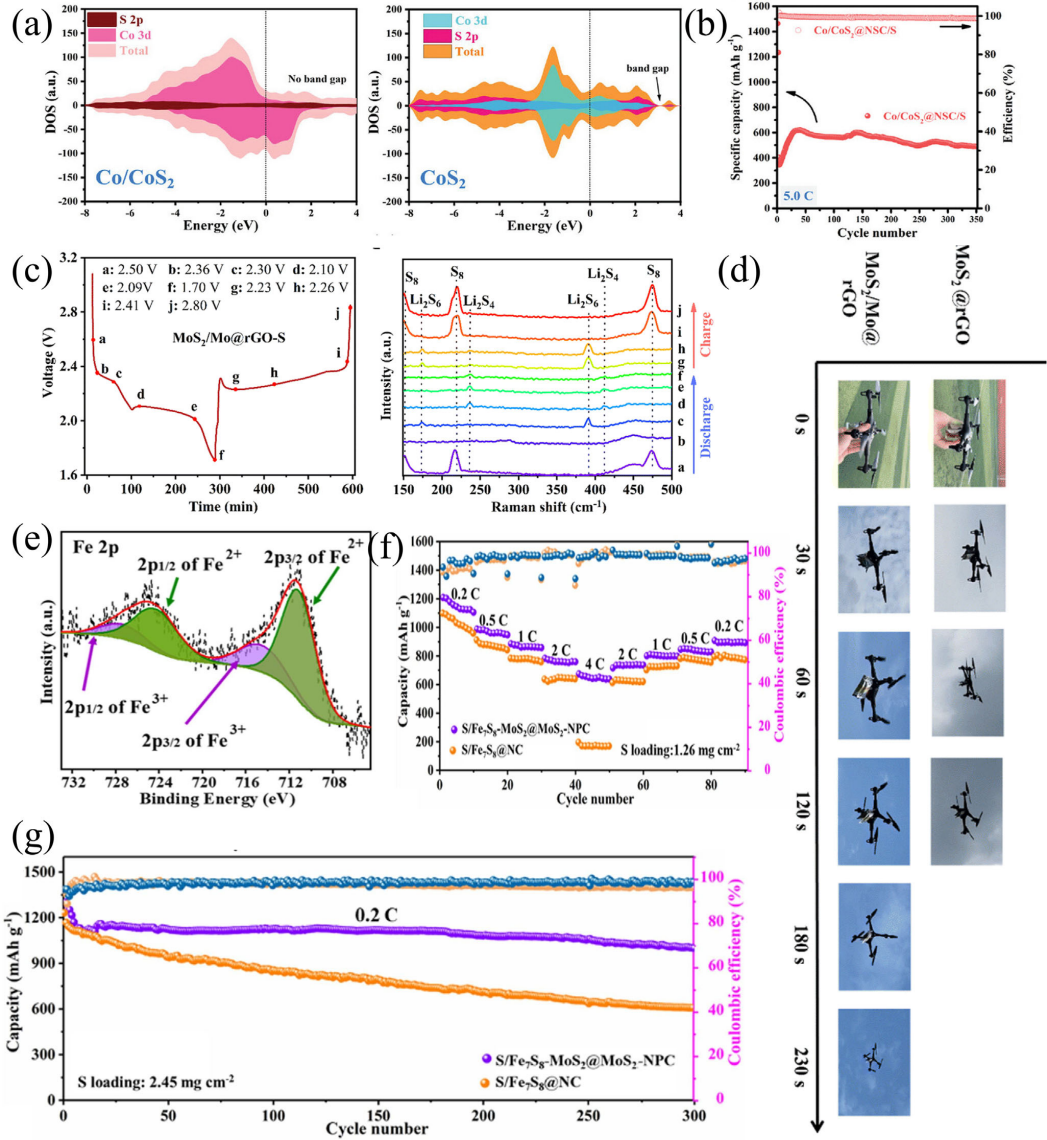
a) The calculated DOS for the Co/CoS_2_, CoS_2_. Reproduced with permission.^[^ ^106^ ^]^ Copyright 2023, Wiley‐VCH. b) Short cycling performance of Co/CoS_2_@NSC/S at 5.0 C. Reproduced with permission.^[^ ^106^ ^]^ Copyright 2023, Wiley‐VCH. c) Charge–discharge curves and in situ Raman spectra of MoS_2_/Mo@rGO‐S. Reproduced with permission.^[^ ^107^ ^]^ Copyright 2025, Royal Society of Chemistry. d) Flight process of the drone powered by MoS_2_@rGO‐S cells and MoS_2_/Mo@rGO‐S cells. Reproduced with permission.^[^ ^107^ ^]^ Copyright 2025, Royal Society of Chemistry. e) Fe 2p in the Fe_7_S_8_‐MoS_2_@MoS_2_‐NPC composite. Reproduced with permission.^[^ ^108^ ^]^ Copyright 2022, Elsevier. f) Rate capabilities of the S/Fe_7_S_8_‐MoS_2_@MoS_2_‐NPC cells. Reproduced with permission.^[^ ^108^ ^]^ Copyright 2022, Elsevier. g) Long cycling performance of S/Fe_7_S_8_‐MoS_2_@MoS_2_‐NPC and S/Fe_7_S_8_@NC electrodes at 1 C. Reproduced with permission.^[^ ^108^ ^]^ Copyright 2022, Elsevier.

Heterostructure is also an effective modification method to enhance the performance of functional materials. By combining two materials with different characteristics, a heterogeneous interface is formed at the junction, where the density of electronic states is enhanced through electron rearrangement, thereby improving the conductivity of the material. For example, Zheng et al.^[^ ^106^ ^]^ prepared Co/CoS_2_ heterostructure loaded in N, S co‐doped carbon nanocages by sulfidizing and carbonizing CoZn‐ZIF precursors (Co/CoS_2_@NSC). Due to the difference in electronic density of states between metallic cobalt and CoS_2_, the electronic density of states at the heterogeneous interface is higher after their interaction, and the overall electronic density of Co/CoS_2_ material is higher than that of CoS_2_ material (**Figure** **9**a), indicating that the intrinsic conductivity of Co/CoS_2_ material is higher, which provides a faster electron transfer rate in sulfur redox reactions. The utilization of carbon nanocages endows the Co/CoS_2_@NSC host material with a high specific surface area of 128.91 m^2^ g^−1^, which not only guarantees a high sulfur content in the cathode but also improves the structural stability. Therefore, in the long‐term cycling test of the battery with Co/CoS_2_@NSC/S as the cathode at a rate of 1 C, the Co/CoS_2_@NSC/S cell demonstrated an initial discharge specific capacity of 1054 mAh g^−1^. Remarkably, it could sustain a reversible discharge capacity of 675 mAh g^−1^ after 500 cycles, with an average capacity decay rate of merely 0.072% per cycle. Furthermore, even in the high‐rate cycling test at 5 C, the Co/CoS_2_@NSC/S battery could still deliver a high capacity of 620 mAh g^−1^ and exhibited outstanding stability over 300 cycles (Figure 9b). Similarly, Cheng and colleagues employed the hydrothermal method to synthesize Mo/MoS_2_ heterostructures on oxidized graphene.^[^ ^107^ ^]^ In this composite, graphene serves as a conductive network for electron transport, while metallic Mo atoms offer supplemental electron transfer channels. This synergistic effect enhances the material's electrical conductivity and facilitates the conversion of polysulfides. As revealed by cyclic voltammetry discharge curves coupled with in situ Raman spectroscopy (Figure 9c), no Li_2_S_8_ or Li_2_S_2_ signal peaks were detected in the Raman spectra corresponding to the two discharge plateaus (b,c and d,e) and the charge plateau (g–i). This indicates that the Mo/MoS_2_@rGO‐S electrode accelerates polysulfide conversion, thereby shortening the lifetime of long‐chain LiPSs and mitigating the shuttle effect between the cathode and anode. Even under high sulfur loadings of 3 and 4 mg cm^−2^, the MoS_2_@rGO‐S batteries can still achieve high initial discharge specific capacities of 1160 and 1026 mAh g^−1^ at a rate of 2 C, respectively and the capacities are maintained at 543 and 480 mAh g^−1^ after 500 cycles. Moreover, this composite material showed great promise for practical applications. As an illustration, the Mo/MoS_2_@rGO‐S pouch battery was proven to be capable of powering a small drone, enabling it to fly steadily for 230 s (Figure 9d).

Numerous groundbreaking studies have shown that 2D MoS_2_ nanosheets are promising LiPSs fixatives and catalysts. However, the poor electronic conductivity and limited catalytic sites of MoS_2_ may lead to a reduced utilization efficiency of sulfur, consequently undermining its overall performance in LSBs. To fundamentally modulate the band structure of MoS_2_, Li et al.^[^ ^108^ ^]^ developed a Fe_7_S_8_‐MoS_2_ heterostructure modified with carbon nano capsules as an effective catalyst for lithium sulfur batteries. The Fe_7_S_8_‐MoS_2_ heterostructure remarkably improves the adsorption and catalytic functions toward lithium polysulfides. Specifically, magnetite Fe_7_S_8_, characterized by the co‐existence of Fe (II) and Fe (III) in mixed valence states (Figure 9e), displays inherent metallic properties, which ensures rapid electron transfer. Furthermore, once the heterojunction is formed, there is an increase in the catalytic sites available for polysulfides. As a result, the conversion kinetics of LiPSs are expedited, which is highly favorable for realizing high‐rate performance (Figure 9f). Based on the above advantages, batteries using this catalyst as the positive electrode exhibit a high specific capacity of 1250.5 mAh g^−1^ under high load conditions (Figure 9g). Similarly, Xu et al.^[^ ^109^ ^]^ incorporated the Co_9_S_8_‐MoS_2_ core‐shell heterostructure with carbon nanofibers. This composite structure offers a double advantage. On the one hand, it promotes the adsorption and conversion of LiPSs. On the other hand, it enhances the electrical conductivity. As a result, even under a high sulfur loading of 6 mg cm^−2^, it demonstrates a high discharge specific capacity of 1002 mAh g^−1^.

#### 2.4.2 Construct Built‐In Electric Fields

**Figure 10:**
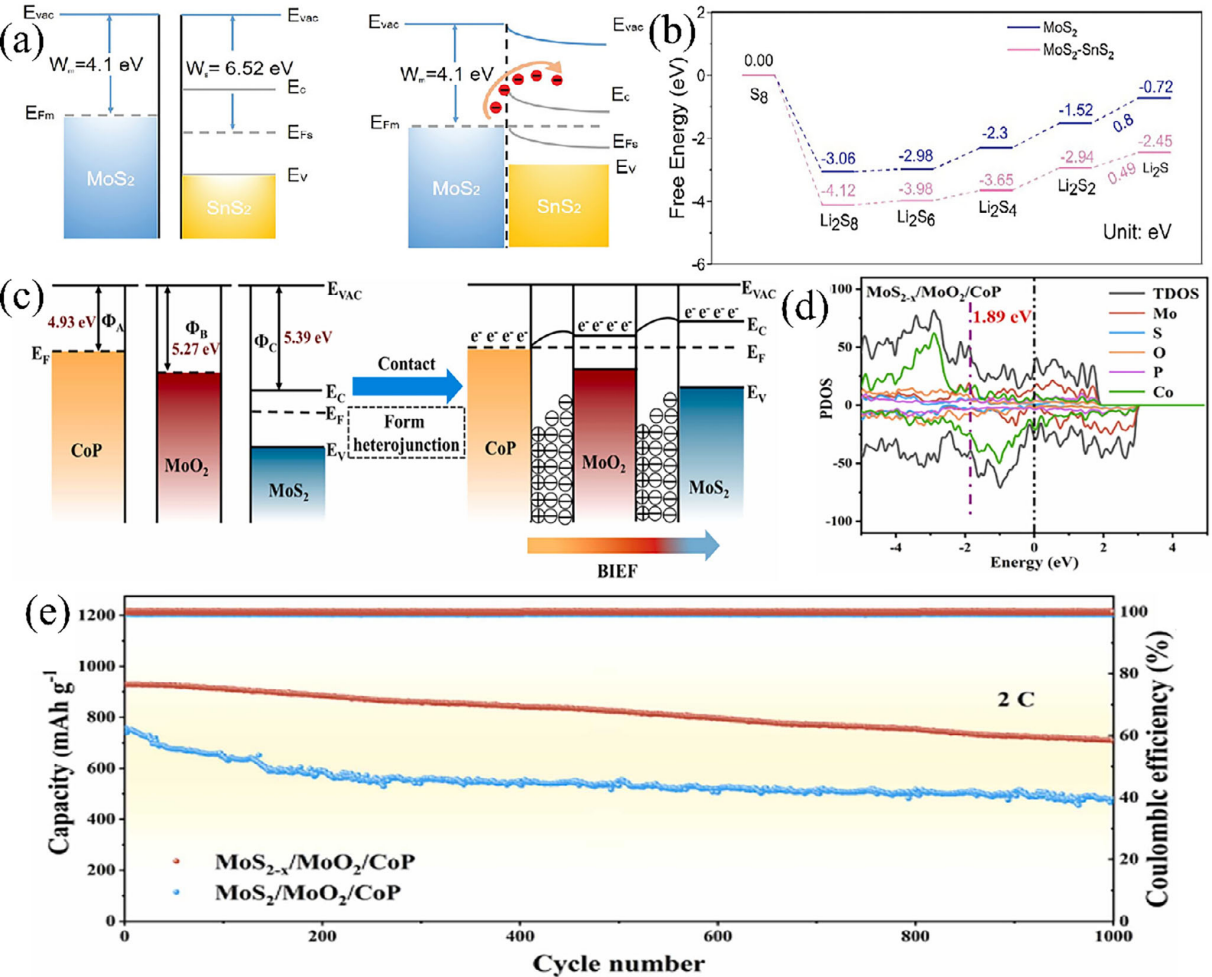
a) Schematic of energy band changes in composites before and after ohmic contacts. Reproduced with permission.^[^ ^112^ ^]^ Copyright 2024, Wiley‐VCH. b) Free energy evolution of LiPSs on reduced MoS_2_ (001) and MoS_2_‐SnS_2_ (001) surfaces. Reproduced with permission.^[^ ^112^ ^]^ Copyright 2024, Wiley‐VCH. c) Energy‐band diagrams of MoS_2‐x_/MoO_2_/CoP ternary heterojunction before and after contact formation of the built‐in electric field. Reproduced with permission.^[^ ^114^ ^]^ Copyright 2025, Elsevier. d) Projected PDOS curves for MoS_2‐x_/MoO_2_/CoP. Reproduced with permission.^[^ ^114^ ^]^ Copyright 2025, Elsevier. e) Long‐term cycle performance at 2 C for 1000 cycles. Reproduced with permission.^[^ ^114^ ^]^ Copyright 2025, Elsevier.

In terms of solid‐state theory, when a metal comes into contact with a semiconductor (taking N‐type semiconductors as an example), electrons will spontaneously flow from the metal to the semiconductor due to the difference in their work functions (Φm < Φs), reducing unnecessary losses and increasing the efficiency of electron migration.^[^ ^110^ ^]^ Two sulfides with different work functions can also form heterogeneous interfaces similar to Ohmic contacts. This Ohmic contact can make the migration direction of electrons more uniform, thereby improving the efficiency of charge transfer and promoting the redox kinetics of sulfur more quickly, thus improving the performance of the battery.^[^ ^111^ ^]^ For example, Chen et al.^[^ ^112^ ^]^ synthesized 1D MoS_2_‐SnS_2_ heterostructures on carbon nanotubes using a two‐step hydrothermal method. Since the work function of MoS_2_ is smaller than that of SnS_2_, electrons will migrate directionally from MoS_2_ to SnS_2_ after the formation of the heterostructure (**Figure** **10**a). Compared to MoS_2_ and SnS_2_, the electronic density of states of MoS_2_‐SnS_2_ hetero‐junction material is higher, and the d‐orbital center has also shifted upward. As shown in Figure 10b, the calculation results show that the Gibbs free energy (0.8 eV) for generating Li_2_S on MoS_2_ is greater than that on MoS_2_‐SnS_2_ (0.49 eV), indicating that LiPSs are more easily reduced on MoS_2_‐SnS_2_. In addition, the decomposition energy (0.21 eV) of Li_2_S on MoS_2_‐SnS_2_ heterojunction is also lower. Therefore, during the long‐term cycling tests conducted at rates of 1 C and 2 C, the CNT‐MoS_2_‐SnS_2_ batteries reach specific capacities of ≈900 and 800 mAh g^−1^, and the capacity retention rates are still 84.8% and 83% after 500 cycles, respectively, demonstrating excellent cycling performance. Sulfides featuring distinct atomic ratios have the capacity to establish built‐in electric fields (BIEF), which play a crucial role in facilitating electron transfer. Xing et al.^[^ ^113^ ^]^ proposed a twin CoS_2_/CoS_1.079_ heterojunction encapsulated in N‐doped hollow carbon polyhedra and in situ generated carbon nanotubes (CoS_2_/CoS_1.079_@NC/CNT), which was used as an electrocatalyst for the sulfur cathode. Its hollow structure is beneficial for sulfur loading. Moreover, the heterojunction offers an abundance of chemisorption sites, facilitating the formation of chemical bonds with polysulfides and enabling the efficient adsorption of LiPSs. More significantly, the spontaneously generated BIEF at the CoS_2_/CoS_1.097_ interface acts as a driving force and convenient channel for electron transfer, thereby promoting the reversible conversion of sulfur. Therefore, the LSBs with the cathode exhibit a high discharge capacity of 1454.4 mAh g^−1^ at 0.1 C and excellent long‐term cycling stability capacity at a high rate of 2 C, with a decay rate of 0.054%.

By pairing adsorbents of high adsorption and high work function with catalysts of moderate adsorption, high catalytic activity, and low work function, and subsequently introducing a kind of catalyst with an intermediate work function and excellent electronic conductivity, a heterogeneous structure that can automatically generate a BIEF is formed. This structure plays a significant role in promoting uniform electron transfer and tuning the d‐band.^[^ ^115^ ^]^ Taking the ternary heterostructure MoS_2‐x_/MoO_2_/CoP as an example,^[^ ^114^ ^]^ MoS_2‐x_ featuring sulfur vacancies demonstrates a strong adsorption capacity and a relatively high work function, yet its catalytic activity remains limited. CoP has a moderate physical adsorption effect and a strong chemical interaction with lithium polysulfides, but its work function is low. MoO_2_, whose properties lie in the intermediate range, can serve as a connecting bridge between the two catalysts and exhibits work function characteristics that are intermediate between those of MoS_2‐x_ and CoP, thereby fulfilling a buffering function. As a result, an interface BIEF was formed (Figure 10c), which significantly facilitated the directional movement of LiPSs, guiding them from the side (MoS_2‐x_) where they were strongly adsorbed toward the side (CoP) with high catalytic efficiency. In addition, DFT calculations have shown that upon the formation of the heterostructure, the electronic and intrinsic properties of the material transform from semiconductor‐like to metallic, which brings the d‐band center more proximate to the Fermi level (Figure 10d). Based on the d‐band center theory, higher d‐band center energy levels in metals lead to an increase in the filling of antibonding bands, thereby enhancing the stability and strength of adsorption bonds. This heterostructure had strong catalytic and adsorption capabilities for polysulfides, thereby accelerating the redox kinetics of sulfur and improving the performance of LSBs. At a rate of 2 C, the battery using this catalyst exhibited a high discharge specific capacity of 930.6 mAh g^−1^ in the first cycle, and then sustained a low‐capacity decay rate of 0.02% per cycle during 1000 cycles (Figure 10e).

#### 2.4.3 Regulate p and d Orbitals

**Figure 11:**
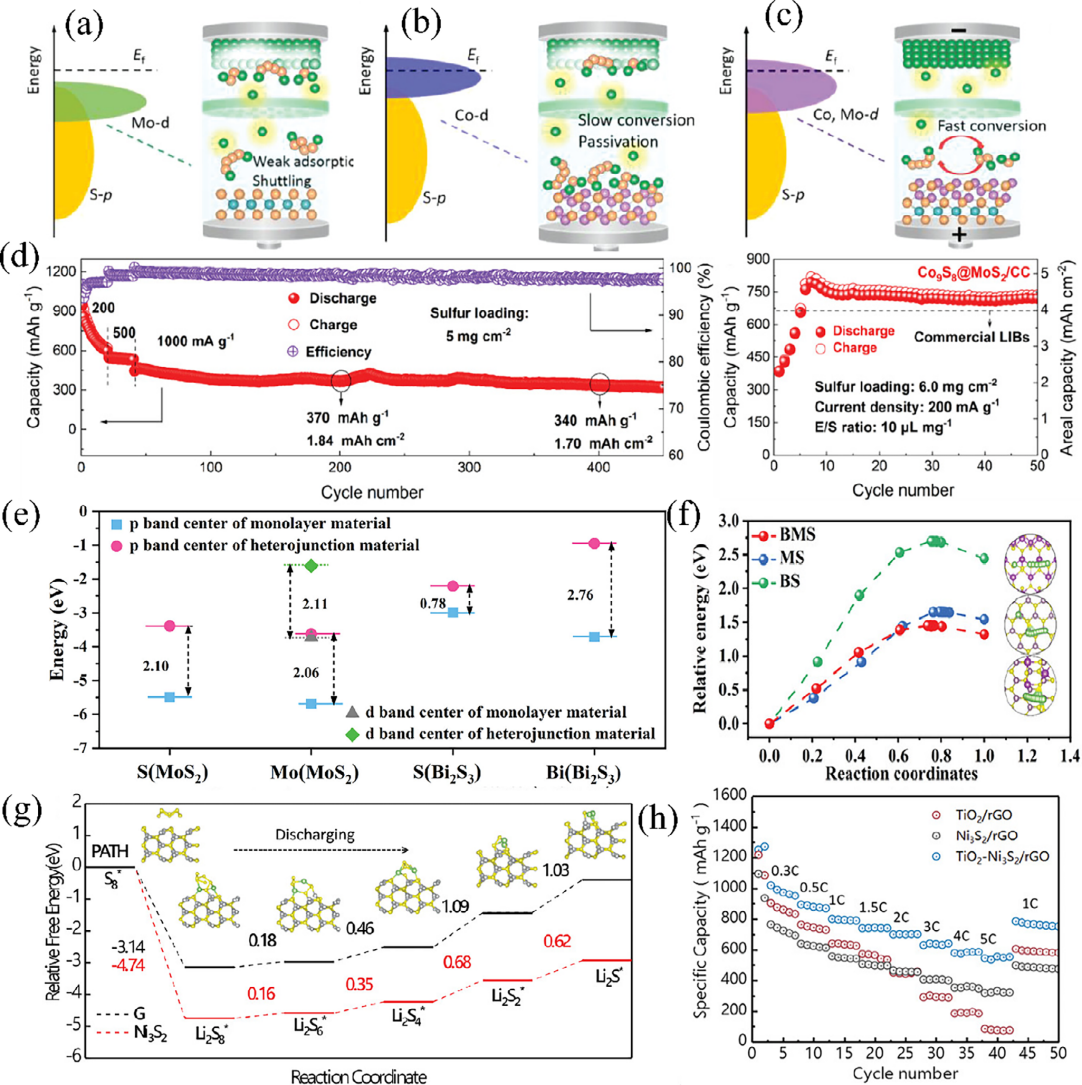
Schematic illustration of the d‐p band center and LiPSs conversion process on a) MoS_2_ catalyst surfaces, b) Co_9_S_8_ catalyst surfaces, and c) Co_9_S_8_@MoS_2_ catalyst surfaces. Reproduced with permission.^[^ ^116^ ^]^ Copyright 2022, Wiley‐VCH. d) Long cycling stability and corresponding Coulombic efficiency of the LSB with Co_9_S_8_@MoS_2_ host at 1000 mA g^−1^ and cycling capacity of Co_9_S_8_@MoS_2_/CC battery under the mass loading of sulfur at 6.0 mg cm^−2^. Reproduced with permission.^[^ ^116^ ^]^ Copyright 2022, Wiley‐VCH. e) Changes of p‐band and d‐band center of MS and BS before and after heterostructure formation. Reproduced with permission.^[^ ^117^ ^]^ Copyright 2024, Wiley‐VCH. f) Dissociation paths and dissociation energy changes of Li_2_S on different materials. Reproduced with permission.^[^ ^117^ ^]^ Copyright 2024, Wiley‐VCH. g) Energy profiles for the reduction of sulfur and LiPSs on graphene and Ni_3_S_2_ (010) surface. Reproduced with permission.^[^ ^119^ ^]^ Copyright 2020, Wiley‐VCH. h) Rate performance of the cells with these samples at different current densities. Reproduced with permission.^[^ ^119^ ^]^ Copyright 2020, Wiley‐VCH.

Besides the advantages mentioned above, heterostructures are capable of regulating the d‐orbital centers of metal atoms to improve the adsorption energy and catalytic effect on intermediate polysulfides, thereby accelerating the conversion of LiPSs and suppressing shuttle effects. For example, Guo et al.^[^ ^116^ ^]^ designed the Co_9_S_8_@MoS_2_ heterogeneous structure, which plays a crucial role in promoting the kinetics of the sulfur reduction reaction/sulfur precipitation reaction (SRR/SER) and in suppressing the shuttle of polysulfides. Theoretical calculations show that the differences between the d‐band centers of the three structures and the Fermi level are −2.13 eV (Co_9_S_8_@MoS_2_), −2.88 eV (MoS_2_), and −1.77 eV (Co_9_S_8_), respectively. The relatively low energy position of d‐band center in MoS_2_ results in a weak interaction between it and polysulfides (**Figure** **11**a). In contrast to the situation of MoS_2_, the Co‐d band center of Co_9_S_8_ is very near to the Fermi level (Figure 11b), indicating a stronger interaction with the electron orbitals of LiPSs. This strong interaction causes the deactivation of active sites and the retardation of conversion kinetics. For the Co_9_S_8_@MoS_2_ heterostructure (Figure 11c), the position of the d‐orbital center is between those of the above two materials, enabling the anchor and decomposition of LiPSs in the vicinity of the surface area to be feasible. In short, the constructed Co_9_S_8_@MoS_2_ heterostructure is a performance‐optimized catalyst obtained from the modification of Co_9_S_8_ and MoS_2_ through electronic orbital regulation, with moderate adsorption energy and enhanced SRR kinetics of LiPSs. Therefore, even under high sulfur loading conditions, the battery can exhibit relatively stable cycling performance (Figure 11d). Similarly, the Bi_2_S_3_‐MoS_2_ (BMS) heterostructure synthesized by Li et al.^[^ ^117^ ^]^ via a multi‐step hydrothermal approach demonstrates a pronounced tuning effect on the p‐band center of sulfur and the d‐band center of molybdenum. Specifically, subsequent to the formation of the heterostructure, it induces the migration of the d‐band center of Mo within MoS_2_ and the p‐band centers of Bi and S within Bi_2_S_3_ toward the Fermi level, as depicted in Figure 10e. This alteration endows BMS with a more robust interaction with LPSs. Consequently, diverse polysulfides exhibit higher adsorption energies on the BMS heterostructure in comparison to monolayer Bi_2_S_3_ and MoS_2_. Furthermore, the decomposition energy of Li_2_S is diminished to 1.45 eV (Figure 11f). This clearly indicates that the catalyst material can effectively facilitate both the charging and discharging processes of Li–S batteries. The LSBs employing this heterostructure as a catalyst also showcase outstanding rate performance. They are capable of attaining an initial specific capacity of 634.7 mAh g^−1^ at a current rate of 3 C. After 500 cycles, they still retain a high reversible specific capacity of 446.7 mAh g^−1^. Furthermore, enhancing catalytic performance can also be achieved by adjusting the position of Ep (the peak closest to the Fermi level). This is because a higher Ep position leads to a lower occupancy of antibonding orbitals, resulting in stronger interactions between LiPSs and the catalyst. Li et al.^[^ ^118^ ^]^ prepared Co_3_S_4_/MnS heterostructures with nano tubular arrays grown on carbon cloth as sulfur cathodes. Subsequent first principles calculations showed that the introduction of Mn regulated the electronic structure of Co_3_S_4_, causing a shift in the Ep position in the electronic density of states of Co_3_S_4_. This shift enhanced the interaction with polysulfides and promoted charge transfer, which was more conducive to the S and Co bonding of Li_2_S_6_, thereby improving the conversion kinetics. The electrochemical performance test results show that CC@CMS‐NA‐based positive electrode was able to maintain 95% of its capacity after 200 cycles at a current density of 2.67 mA cm^−2^, demonstrating excellent cycling stability.

The ability of heterostructures to regulate the electronic states (electron density of states and band positions) of sulfides is not limited to this. Combining sulfides with more polar oxides to form heterostructures can also regulate the electronic state, thereby achieving appropriate catalytic performance of the material. For instance, Wang et al.^[^ ^119^ ^]^ conceived and synthesized the TiO_2_‐Ni_3_S_2_ heterostructure via a straightforward hydrothermal approach. Given the pronounced polarity of the two materials, upon the formation of the heterojunction interface between TiO_2_ and Ni_3_S_2_, the p‐orbitals of the S atoms in Ni_3_S_2_ and the d‐orbitals of the Ti atoms in TiO_2_ unavoidably overlap, thereby forming hybrid orbitals. The charge density difference further reveals that substantial charge transfer and redistribution take place at the interface of TiO_2_‐Ni_3_S_2_. Owing to this orbital hybridization, the interactions between heterostructures and polysulfides are significantly enhanced, thereby reducing the free energy required for the decomposition of various sulfur species during the discharge process (as illustrated in Figure 11g). Notably, TiO_2_‐Ni_3_S_2_ concurrently exhibits dual functions of catalysis and adsorption. Specifically, the highly polar TiO_2_ captures LiPSs during the reduction process. Subsequently, the liquid polysulfides diffuse across the interface to Ni_3_S_2_, which possesses stronger catalytic capabilities, enabling rapid conversion. This systematic and highly efficient conversion mechanism considerably shortens the lifespan of intermediate products, curbs the shuttle effect, and concurrently averts the deactivation of the catalyst surface caused by coverage. This characteristic endows the cathode with high stability and activity, thereby enabling it to exhibit excellent rate performance (Figure 11h). Consequently, the sulfur cathode incorporating TiO_2_‐Ni_3_S_2_ as the catalyst displayed a capacity decay of 0.038% after 900 cycles at a 0.5 C rate, and it retained a capacity retention rate of 65% after 500 cycles at a 0.3 C rate with a sulfur loading of 3.9 mg cm^−2^. This study reveals how bidirectional catalysts can facilitate the practical implementation of LSBs. Bifunctional heterostructures with the ability to regulate the p‐and d‐bands have also been constructed in the latest research results, and it has been proven that the heterostructures play a crucial role in improving the performance of LSBs, such as 1T‐MoS_2_/C_3_N_4_^[^ ^120^ ^]^ and CoSe_2_@CoS_2_/NC.^[^ ^121^ ^]^

#### 2.4.4 Other Types of Heterostructures

So far, numerous heterostructure composite materials have been systematically investigated, particularly those composed of transition metal sulfides featuring either identical anions and distinct metal ions (MoS_2_/Ni_3_S_2_) or identical metal ions and different anions (Fe_9_S_10_/Fe_3_O_4_).^[^ ^122^, ^123^ ^]^ For transition metal sulfide‐based heterostructures incorporating diverse anionic and cationic components, a two‐step synthesis approach is typically adopted. In this method, one component is synthesized initially, followed by the controlled growth of another metal compound on its surface. When dealing with heterostructures that contain identical metal ions yet different anions, the initial step could be to prepare a single‐component metal compound. Then, the heterostructure can be obtained by adjusting the soaking time, sintering temperature and atmosphere. Alternatively, it can be prepared by sintering with a chemical reagent containing the target anion at high temperatures. For example, thiourea is added to prepare a sulfide heterojunction. By adjusting the degree of sulfidation of WO_3_, a balance between the trapping ability of WO_3_ and the catalytic ability of WS_2_ in the WS_2_‐WO_3_ heterostructure is achieved.^[^ ^124^ ^]^ Among them, the LSB with a 3WO_3_:1WS_2_ ratio attains the highest conversion efficiency. It is evident that the preparation process of sulfides directly determines the structure of the catalyst material, which in turn indirectly affects the performance of the battery. Beyond this, when one constituent of a heterostructure exhibits superior adsorption capabilities and another demonstrates exceptional catalytic activity, the adsorption‐diffusion‐catalysis process of polysulfides is significantly improved at the heterostructure interface. This seamless process effectively promotes redox kinetics, as evidenced by the MoS_2_‐MoN.^[^ ^125^ ^]^ Heterostructure composite materials offer a dual advantage. They not only amalgamate the favorable properties of individual materials but also harness interface‐related effects. These effects include expediting charge transfer, facilitating the diffusion of Li^+^ ions and LiPSs, and enhancing the overall adsorption capacity. Undoubtedly, heterostructures have a pronouncedly positive impact on advancing the commercialization of Li–S cells.

Nevertheless, they are not without drawbacks. When a heterostructure incorporates a large number of components, the reaction mechanism within lithium‐sulfur batteries becomes highly convoluted and arduous to decipher. This complexity poses a significant hurdle to further enhancing the performance of the materials. Given that each component contributes distinct functions, finding an effective way to balance the competing characteristics of various sulfides and maximize the potential of each component is a pressing challenge that demands immediate attention. Looking ahead, future research endeavors should intensify efforts in two key areas. First, a deeper exploration of the reaction mechanisms is essential to gain a comprehensive understanding of the underlying processes. Second, there is a need to innovate and develop a wider array of heterostructure types, such as multi‐element heterostructures, multi‐component heterostructures, and hetero‐atom‐doped heterostructures, which will not only expand the scientific knowledge but also open up new possibilities for improving the performance of LSBs.

### 2.5 Composite Materials of TMS and MXene/MOF

#### 2.5.1 MXene‐Based TMS Composite Materials

MXenes are a new type of 2D layered transition metal carbide or nitride. The initial generation of MXenes, specifically Ti_3_C_2_T_x_ with T signifying O, OH, or F, was created via the removal of the A layer within the MAX phase by means of etching. MAX follows the general formula M_n+1_AX_n_. In this formula, M corresponds to early transition metals. A predominantly belongs to transition metal groups including elements like Mo, Nb, Ti, V, and so on. X represents either C or N, and n takes values ranging from 1 up to 3.^[^ ^126^ ^]^ The new material has excellent metal conductivity, unique mechanical properties, adjustable interlayer spacing, and abundant surface termination groups, which enhance electron/ion transport, improve sulfur utilization and make it a promising candidate for sulfur positive electrodes when integrated into the cathode of Li–S batteries.^[^ ^127^ ^]^ Nevertheless, due to the existence of van der Waals interactions and hydrogen bonding, MXene nanosheets tend to restack readily. This restacking phenomenon leads to a significant reduction in the number of active sites. Moreover, MXenes exhibit poor affinity for sulfur, which further impedes the adsorption of polysulfides. In addition, the limited electrocatalytic capabilities of MXenes are insufficient to promote the conversion of LiPSs and Li_2_S, as well as the associated reaction kinetics. Consequently, modifying MXenes to enhance their surface polarity is imperative to strengthen its sulfur immobilization ability. Composite materials of sulfides and MXenes have been fabricated. The addition of sulfides can reduce the stacking degree of MXene sheets and expose the active sites simultaneously, thus improving the overall performance.

**Figure 12:**
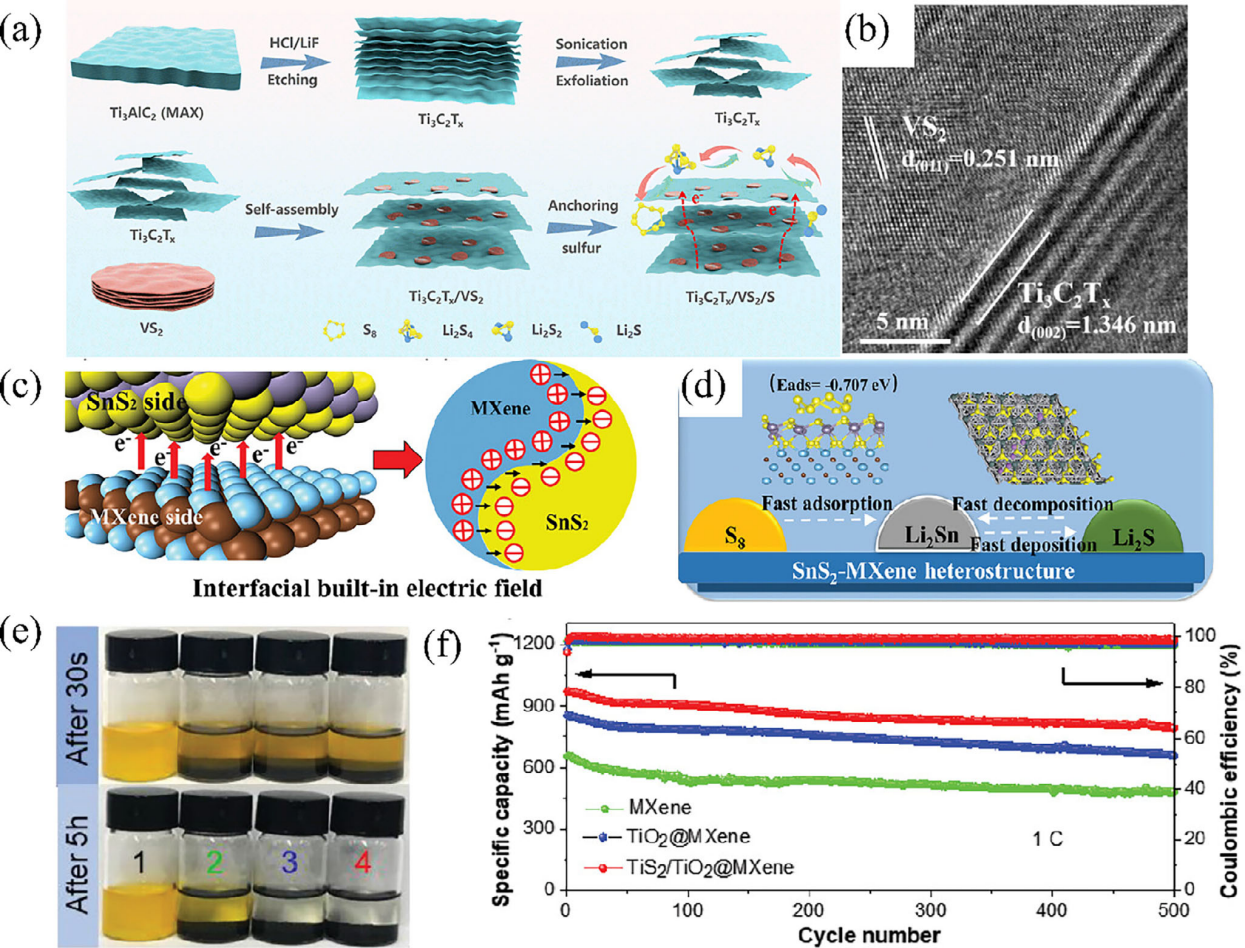
a) Schematic illustration of the synthesis of Ti_3_C_2_T_x_/VS_2_ and the LiPSs conversion mechanism. Reproduced with permission.^[^ ^128^ ^]^ Copyright 2024, Royal Society of Chemistry. b) HRTEM images of Ti_3_C_2_T_x_/VS_2_. Reproduced with permission.^[^ ^128^ ^]^ Copyright 2024, Royal Society of Chemistry. c) Schematic illustration of the electron flow direction simulation and interfacial BIEF. Reproduced with permission.^[^ ^129^ ^]^ Copyright 2023, Wiley‐VCH. d) LiPSs conversion on MXene‐SnS_2_ surfaces. Reproduced with permission.^[^ ^129^ ^]^ Copyright 2023, Wiley‐VCH. e) Optical photographs of the visual Li_2_S_6_ adsorption test. Reproduced with permission.^[^ ^130^ ^]^ Copyright 2023, Wiley‐VCH. f) Long‐term cycling stability of different cells at 0.1 C and 1 C, respectively. Reproduced with permission.^[^ ^130^ ^]^ Copyright 2023, Wiley‐VCH.

For instance, Deng et al.^[^ ^128^ ^]^ adopted a special one‐step hydrothermal synthesis technique to fabricate sheet shaped titanium carbide (Ti_3_C_2_T_x_) materials and VS_2_ nanosheets, separately. Subsequently, these materials underwent self‐assembly driven by van der Waals forces, leading to the generation of Ti_3_C_2_T_x_/VS_2_ composite materials (**Figure** **12**a). This self‐assembly mechanism enables the attachment of VS_2_ nanosheets onto the surface of titanium carbide sheets (Figure 12b), ultimately constructing a bifunctional catalyst structure. The configuration simultaneously enhances electronic conductivity, mitigates electrode expansion, and improves the cycling stability of composite electrodes. Even the oxidation and stacking phenomena of titanium carbide under high temperature conditions are significantly prevented by the heterostructure. Leveraging these advantages, the Ti_3_C_2_T_x_/VS_2_ material exhibits outstanding electron transfer ability when employed as a sulfur host material, robust adsorption and anchoring effects on lithium polysulfides, and superior electrocatalytic activity, effectively mitigating the polysulfides shuttle effect. To further enhance conductivity, carbon nanotubes (CNTs) were incorporated as conductive binders in the sulfur cathode, replacing traditional polymer binders. The resulting Ti_3_C_2_T_x_/VS_2_/S composite cathode achieved an initial discharge capacity of 1382.7 mAh g^−1^ at 0.1 C, retaining a reversible capacity of 806.6 mAh g^−1^ after 150 cycles. Additionally, a 3D grid electrode architecture with high sulfur loading (10.05 mg cm^−2^) was successfully fabricated using 3D printing technology with an area specific capacity maintained at 7.73 mAh cm^−2^ after 100 cycles at 0.05 C, demonstrating its potential for practical high‐energy‐density applications.

Similar to heterostructures, a built‐in electric field can also be formed between MXene and TMSs, thereby improving the electrical properties of the materials. Chen et al.^[^ ^129^ ^]^ successfully constructed a multifunctional MXene‐based (Ti_3_C_2_T_x_) heterostructure with SnS_2_ via a facile hydrothermal synthesis, creating an inter‐facial built‐in electric field to simultaneously enhance sulfur adsorption and electrocatalytic performance. Comprehensive characterization combining DFT calculations, dynamic electrochemical measurements, and microstructural analysis revealed that the MXene facilitates electron loss from Ti atoms, while the SnS_2_ matrix exhibits strong electron‐accepting behavior through its S atoms, which indicates that the SnS_2_ semiconductor phase possessed a higher work function compared to the MXene component (equivalent to metallic phases). Therefore, upon the contact between MXene and SnS_2_ to form a heterostructure, electrons from Ti atoms gradually migrate to the adjacent S atoms in SnS_2_ until thermodynamic equilibrium is achieved (Mott‐Schottky effect). This process ultimately results in a depletion of the charge density around Ti atoms in MXene (the surfaces of Ti atoms in MXene carry positive charges), while the charge density of adjacent S atoms in SnS_2_ accumulates (the adjacent S surfaces in SnS_2_ bear negative charges), thereby leading to the formation of a built‐in electric field (as depicted in Figure 12c). The surface of SnS_2_ is rich in electrons, which have a strong tendency to form chemical bonds with more LiPSs. This property enables the effective capture of a larger quantity of LiPSs, significantly suppressing the shuttle effect. During Li_2_S nucleation or decomposition, abundant Li^+^ ions and electrons rapidly migrate at the heterojunction, greatly reducing the Li_2_S nucleation/decomposition potential barrier and boosting bidirectional sulfur conversion kinetics (Figure 12d). Finally, the SnS_2_‐MXene heterojunction sulfur cathode with a sulfur loading of 75 wt.% achieved a high reversible capacity of 1188.5 mAh g^−1^ at 0.2 C, and after 500 cycles at 1 C, the capacity decay per cycle was as low as 0.0345%. Importantly, even under sulfur loading of 8.0 mg cm^−2^ and poor electrolyte conditions of 5.0 µL mg^−1^, the SnS_2_‐MXene heterojunction sulfur positive electrode still has a high initial area capacity of 7.35 mAh cm^−2^.

In addition, by combining the strong adsorption properties of oxides and sulfides, Lee et al.^[^ ^130^ ^]^ synthesized a ternary heterostructure (TiS_2_/TiO_2_@MXene) with high‐efficiency adsorption and catalytic performance using MXenes as the matrix. With abundant active sites and similar layered structures, this heterostructure not only has strong adsorption and catalytic abilities for polysulfides, but also can form large interlayer voids to increase the sulfur loading and the energy density of the battery. Subsequently, each sample was placed in a Li_2_S_6_ solution for the adsorption characteristic test. Test tubes No.2, No.3 and No.4 were filled with MXenes, TiO_2_@MXene and TiS_2_/TiO_2_@MXene catalysts respectively. The results shown in Figure 12e indicate that after 5 h of standing, the solutions in test tube No.3 and No.4 have both faded, while the solution in test tube No.2 still remains light yellow. This phenomenon fully demonstrates the strong adsorption effect of the TiS_2_/TiO_2_@MXene composite material on polysulfides, in which TiO_2_ plays a key role. Furthermore, the cyclic voltammetry (CV) curve of the cells with the TiS_2_/TiO_2_@MXene cathode and the distinct redox peaks exhibited in the symmetric cell clearly indicate the outstanding electrocatalytic activity of TiS_2_. Taking these characteristics together, the battery with the S/TiS_2_/TiO_2_@MXene cathode demonstrates excellent cycle stability at a rate of 1 C (Figure 12f). More importantly, the high‐density S/TiS_2_/TiO_2_@MXene cathode achieved a volumetric energy density as high as 2476 Wh L^−1^ under a high sulfur mass loading of 7.5 mg cm^−2^ and a lean electrolyte condition of 5 µL mg^−1^.

An increasing number of analogous composite materials are being developed and employed to drive the advancement of Li–S cells. Moreover, given the substantial environmental harm inflicted by the prevalent synthesis methods for MXenes, it is imperative to explore more efficient modification approaches and eco‐friendly synthesis techniques. These could involve heteroatom doping and low‐temperature hydrothermal methods.

#### 2.5.2 Composites of MOF‐Derived TMSs

In 1995, Yaghi and Li first proposed the concept of metal‐organic frameworks (MOFs), which are formed by connecting metal ions and organic ligands through coordination bonds.^[^ ^131^ ^]^ In recent years, with the development of energy storage devices, MOF materials have demonstrated great application potential in the field of Li–S batteries, thanks to their advantages such as high porosity, excellent adsorption capacity, high structural tunability, and simple synthesis process. For example, the Fe‐based MOFs synthesized by Hornebecq et al. possess a high specific surface area and structural stability, while the Ni‐based MOFs prepared by Cai et al. feature superior conductive frameworks and sulfur affinity—thereby enabling cathodes with high sulfur loading and high specific discharge capacity.^[^ ^132^, ^133^ ^]^ Additionally, in terms of the working mechanism, Zhou et al.^[^ ^134^ ^]^ further demonstrated that compared with the pore size of MOFs, the size of interparticle gaps and the stacking thickness of the MOF layer are more significant factors influencing the modification effect. Furthermore, ultrathin and crack‐free MOF films are suitable for functioning as ion sieves, exerting a selective and homogenizing effect on the migration of polysulfides and lithium ions, thereby enhancing the long‐cycle performance and safety of LSBs.

**Figure 13:**
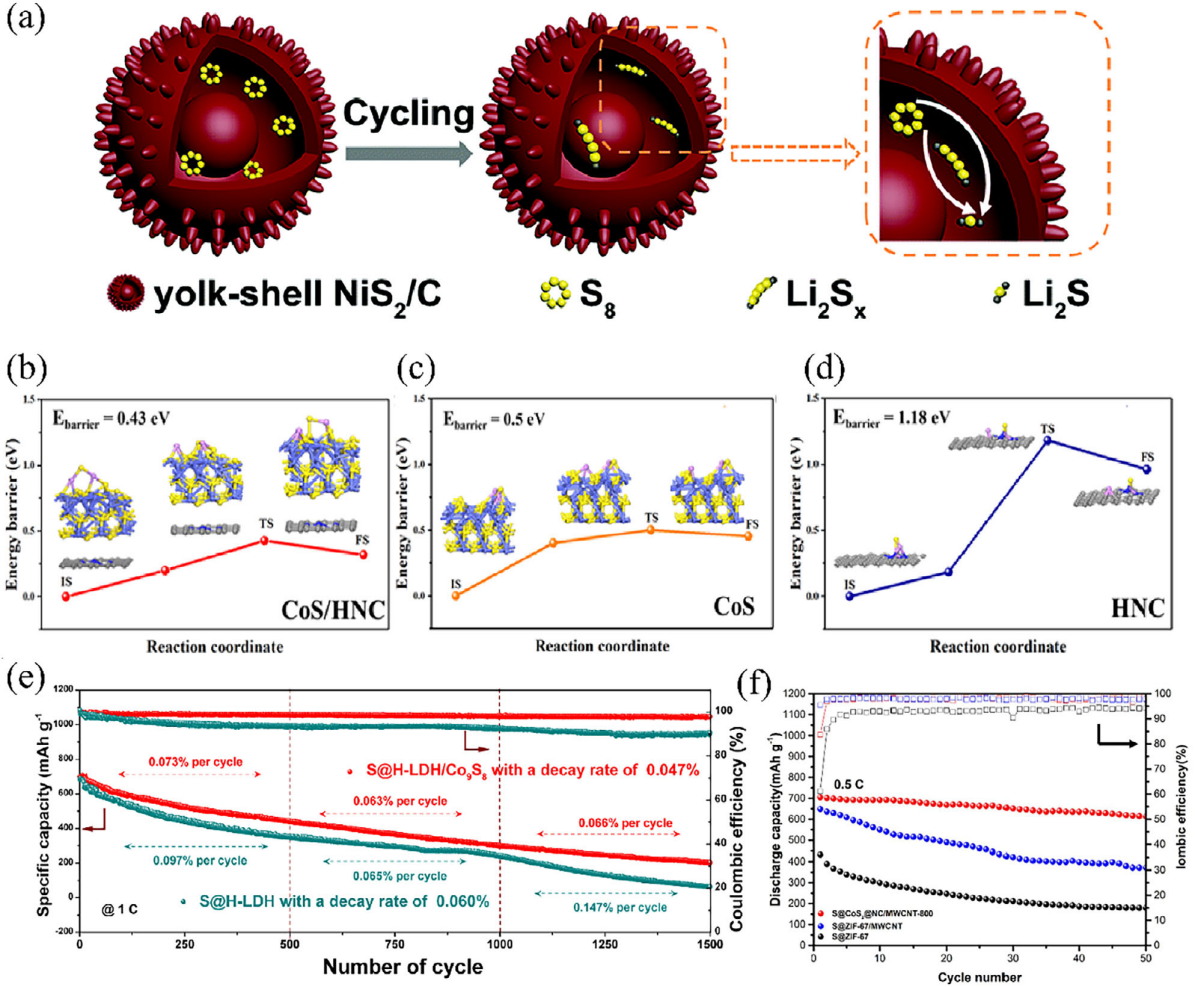
a) Schematic illustration of the advantages of yolk–shell NiS_2_/C–S during the cycling process. Reproduced with permission.^[^ ^136^ ^]^ Copyright 2019, Royal Society of Chemistry. Decomposition energy barriers of Li_2_S on b) CoS/HNC, c) CoS and d) HNC. Reproduced with permission.^[^ ^137^ ^]^ Copyright 2024, Elsevier. e) Long cyclic performance at 1 C of S@H‐LDH and S@H‐LDH/Co_9_S_8_. Reproduced with permission.^[^ ^139^ ^]^ Copyright 2020, Elsevier. f) Cycling stability tests of S@ZIF‐67, S@ZIF‐67/MWCNT, and S@CoS_2_@NC/MWCNT‐800 at 0.5 C. Reproduced with permission.^[^ ^140^ ^]^ Copyright 2022, Elsevier.

However, the presence of insulating organic ligands in MOFs significantly hampers electron transport, resulting in inherently poor electrical conductivity for most MOF‐based materials. Furthermore, the adsorption and catalytic capabilities of pristine MOFs remain inherently restricted, thereby limiting their practical application. Recently, a novel titanium oxide (TiO_2_) based metal‐organic framework (MOF‐TOC) has been reported to effectively restrict lithium polysulfides.^[^ ^135^ ^]^ The active sites within the TOC matrix accelerate the redox processes of sulfur species through d‐p orbital hybridization, thereby substantially enhancing the stability and cycling performance of high‐performance Li–S batteries. Consequently, composite materials formed by integrating sulfides with MOF matrices have demonstrated the ability to overcome the aforementioned limitations while multidimensionally enhancing the overall electrochemical performance. Tian et al.^[^ ^136^ ^]^ converted Ni‐based metal‐organic framework spheres into carbon‐shell‐coated NiS_2_ via the annealing method. This core‐shell structure can confine sulfur and polysulfides within its large internal voids during battery cycling (**Figure** **13**a). More importantly, the derived NiS_2_ is uniformly distributed inside and outside the core‐shell structure, acting as catalytic active sites and thereby enhancing redox kinetics. Benefiting from its unique structure, the NiS_2_/C‐S cathode exhibits lower overpotential and improved cycling stability. Yao et al.^[^ ^137^ ^]^ successfully synthesized a composite material consisting of CoS_2_ and N‐doped hollow carbon (CoS_2_@HNC) as a sulfur host for lithium‐sulfur cells via a facile MOF templated calcination and etching strategy. The hierarchical hollow carbon architecture effectively mitigates sulfur volume expansion during cycling while enabling a high sulfur loading of 74 wt.%. The uniformly distributed CoS_2_ active sites electrocatalytically promote the conversion of LiPSs, as corroborated by theoretical calculations. Moreover, the optimized interface engineering of the composite material modulates the nucleation and deposition mechanism of Li_2_S and reduces the decomposition energy barrier of Li_2_S (Figure 13b–d), thereby preserving the catalytic activity of CoS_2_. At a current density of 0.5 C, the CoS_2_@HNC/S battery initially delivered an outstanding discharge specific capacity of 1015 mAh g^−1^. Impressively, after 100 cycles, the discharge specific capacity remained as high as 883 mAh g^−1^, corresponding to an ultralow average capacity decay rate of only 0.13% per cycle. Even in the 1 C high‐rate long cycle test, the discharge specific capacity remained ≈455 mAh g^−1^ after 800 cycles, with an average decay rate of only 0.05% per cycle.

Zeolite imidazole framework materials (ZIFs), especially ZIF‐8 and ZIF‐67, are two important metal‐organic frameworks (MOFs), which have better stability than other MOF materials.^[^ ^26^ ^]^ In a notable study, Zhang and colleagues employed a composite material comprising MoS_2_ and nitrogen‐doped carbon nanorods derived from ZIF‐67 as a sulfur host.^[^ ^138^ ^]^ The nitrogen‐doped carbon nanorods, stemming from ZIF‐67, create a conducive environment for redox reactions. This structural advantage enables them to effectively accommodate the significant volume changes associated with sulfur during the charge–discharge process. The MoS_2_ coating layer plays a dual‐functional role. Acting as both catalytic and adsorption sites, it facilitates the efficient adsorption and rapid conversion of polysulfides. This mechanism is crucial for mitigating the notorious shuttle effect, a major hurdle in lithium sulfur battery performance. Furthermore, the surface of MOF derived materials is rich in various functional groups. These groups synergistically enhance the material's ability to adsorb and catalyze polysulfides, further weakening the shuttle effect. As a result, the overall performance of the LSBs is substantially improved. Similarly, using ZIF‐67 as a precursor, Chen et al.^[^ ^139^ ^]^ synthesized a nanocage‐like heterostructure composed of Ni/Co‐LDH and Co_9_S_8_. The robust H‐LDH/Co_9_S_8_ host structure serves as a highly effective barrier, significantly curbing the dispersion of polysulfides and mitigating the adverse effects of the volume expansion effect. This exceptional performance can be primarily ascribed to the rich presence of oxygen‐containing functional groups and Co‐S active sites within the structure. These components not only enhance the chemical affinity toward polysulfides but also contribute to the overall stability of the sulfur‐based cathode. Furthermore, the interaction zone between the Ni/Co‐LDH outer shell and the inner Co_9_S_8_ domain plays a pivotal role in promoting charge transfer processes. It effectively boosts electron conductivity and expedites the diffusion of Li^+^ ions, leading to a substantial acceleration of the reaction kinetics involved in the conversion of sulfur species. Owing to these remarkable attributes, the LSBs incorporating this advanced catalyst demonstrate outstanding cycling stability and structural resilience. After 1500 cycles test at 1 C, it retains an impressively high‐capacity retention rate (Figure 13e), highlighting its potential for long‐term and reliable energy storage applications. To improve the electrical conductivity of MOF‐based materials, Chen et al.^[^ ^140^ ^]^ ingeniously combined ZIF‐67 with multi‐walled carbon nanotubes to prepare a composite material (CoS_2_@NC‐MWCNT). Herein, carbon nanotubes provide a convenient channel for electron transfer, while CoS_2_ nanoparticles serve as catalytic and adsorption sites, laying a foundation for the rapid redox reactions of sulfur. Furthermore, the octahedral framework ensures the stability of the sulfur host structure, which will help achieve the long‐cycle performance of LSBs. This catalyst represents a harmonious combination of multiple advantageous properties. When employed as a sulfur host and incorporated into the cathode, the CoS_2_@NC/MWCNTs/S electrode delivers a high specific capacity of 1133 mAh g^−1^ at a current rate of 0.1 C. Even at a rate of 0.5 C, the battery equipped with this catalyst also exhibits an extremely low‐capacity decay rate (Figure 13f).

All in all, these examples underscore the significant potential of metal‐organic frameworks (MOFs) in enabling sulfur utilization within LSBs. To fully exploit their advantages, future research should focus on developing novel MOF‐based composites such as MOF‐MXene heterostructures, single‐atom MOF composites, and dual‐atom MOF composites.^[^ ^26^ ^]^

In conclusion, the four TMS modification methods all enhance electrochemical of performance of LSBs to varying degrees. Finally, the cycling performance of these batteries is summarized in **Table** **2**. From this, doped TMS cathodes achieve high specific discharge capacities, multi‐metallic TMSs and heterostructures ensure long‐term cycling stability, while porous MXene and MOF‐based TMSs boost the sulfur content in the cathode.

**Table 2:** A summary of cycling performance of cathodes for LSBs with TMS catalysts.

Catalysts	Sulfur loading [%]	Current density [C]	Initial‐capacity [mAh g^−1^]	Cycles numbers	Capacity retention [%]	Refs.
ZnS_1‐x_‐CC	‐	1	659	500	79.51	[50]
1T MoS_2_	81	0.05	865	300	83.19	[51]
3%Co‐VS_4_/rGO	73.55	0.2	1332.6	500	55.8	[56]
Mo‐VS_2_/rGO	74.6	1	938.6	500	78.5	[65]
Ni‐WS_2_@rGO	80	0.2	1160.8	100	54.49	[67]
P‐MoS_2_‐G	64	0.5	1106	300	56.9	[75]
Sr_8_Ti_7_S_21_	69.8	0.2	1266	100	94.63	[91]
NiCo_2_S_4‐x_/CNF	65	0.2	922.8	500	71.5	[48]
Ni_0.261_Co_0.739_S_2_@NPCTs	63.6	5	511	1000	45	[94]
Zn_0.30_Co_0.31_Cu_0.19_In_0.13_Ga_0.06_S	60	0.2	706	100	68	[101]
Co/CoS_2_@NSC	52.5	1	1054.4	500	64.02	[106]
Mo/MoS_2_@rGO	64.9	0.3	1309	150	84.2	[107]
CNT‐MoS_2_‐SnS_2_	69.3	2	800	500	83	[112]
CoS_2_/CoS_1.097_@ NC/CNT	74.6	2	827.8	500	73.05	[113]
MoS_2‐x_/MoO_2_/CoP	70	0.2	1356.2	400	75.2	[114]
Co_9_S_8_@MoS_2_	60	‐	820.2	1000	50.1	[116]
Bi_2_S_3_‐MoS_2_	78	3	634.7	500	70.37	[117]
TiO_2_‐Ni_3_S_2_	64	0.5	980	900	65.8	[119]
MoS_2_‐MoN	51	0.2	1100	100	93.9	[125]
Ti_3_C_2_T_x_/VS_2_	49	0.1	1382.7	150	58.34	[128]
SnS_2_‐MXene	75	1	777.3	500	82.7	[129]
TiS_2_/TiO_2_@MXene	72	1	992.59	500	81	[130]
CoS_2_@HNC	80	0.5	1015	100	87	[137]
H‐LDH/Co_9_S_8_	73.4	1	710	1500	29.5	[139]

In summary, owing to their exceptional adsorption and catalytic properties, the integration of various TMS catalysts into cathodes has significantly enhanced the specific discharge capacity, rate capability, and cycling stability of LSBs, yielding promising results. However, research on TMS applications extends beyond this scope. As studies advance, researchers have found that employing TMSs exclusively in cathodes remains insufficient to tackle the fundamental challenge of LSBs—the shuttle effect. Moreover, the formation of lithium dendrites on the lithium anode surface raises concerns regarding battery safety. To address these issues, researchers have proposed novel application strategies for TMSs, including separator modification and the development of composite lithium anodes. These approaches tackle the aforementioned challenges through distinct mechanisms, and these details will be discussed in depth in the subsequent two chapters.

## 3 TMSs for Separator Modification Materials

### 3.1 Tackle the Drawbacks of TMSs for the Sulfur Host Cathode

**Figure 14:**
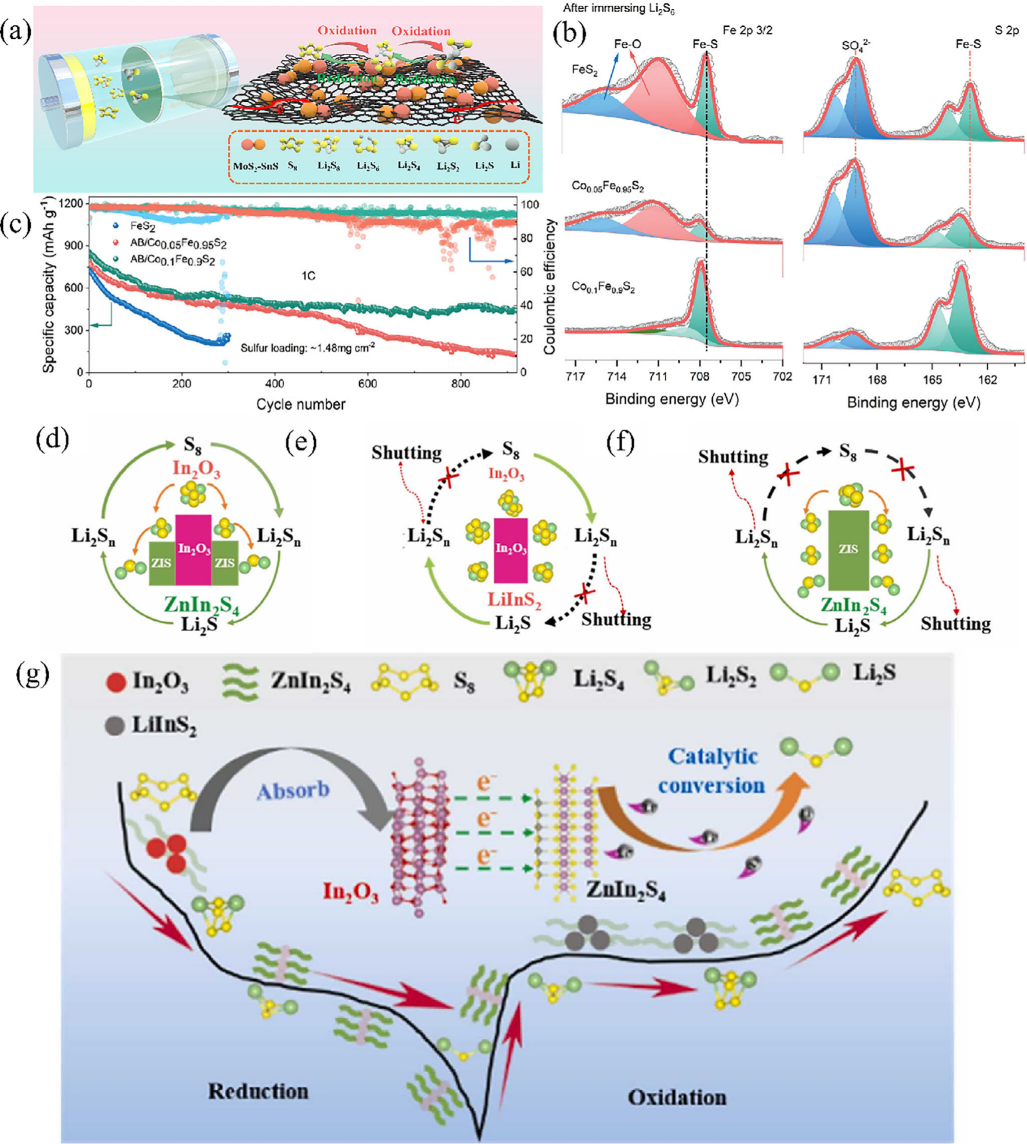
a) Schematic mechanism of the adsorption and conversion of LiPSs on the MoS_2_‐SnS/NC modified separator. Reproduced with permission.^[^ ^142^ ^]^ Copyright 2023, Elsevier. b) XPS fine spectra of Fe 2p^3/2^ and S 2p after Li_2_S_6_ adsorption. Reproduced with permission.^[^ ^143^ ^]^ Copyright 2024, Wiley‐VCH. c) Long cycle performance at 1 C. Reproduced with permission.^[^ ^143^ ^]^ Copyright 2024, Wiley‐VCH. d) Schematic illustrating the catalytic mechanism in Li–S chemistry with ZnIn_2_S_4_ catalysts for tackling the “shuttle effect” of LiPSs. Reproduced with permission.^[^ ^144^ ^]^ Copyright 2024, Elsevier. e) Schematic illustrating the catalytic mechanism in Li–S chemistry with In_2_O_3_ catalysts for tackling the “shuttle effect” of LiPSs. Reproduced with permission.^[^ ^144^ ^]^ Copyright 2024, Elsevier. f) Schematic illustrating the catalytic mechanism in Li–S chemistry with ZIS catalysts for tackling the “shuttle effect” of LiPSs. Reproduced with permission.^[^ ^144^ ^]^ Copyright 2024, Elsevier. g) The calculated free energy values of the discharge process from S_8_ to Li_2_S on different catalysts. Reproduced with permission.^[^ ^144^ ^]^ Copyright 2024, Elsevier.

Employing TMS catalysts as separator‐modifying materials in LSBs is a novel application method in recent years. Unlike their application at the cathode, TMSs on the separator can effectively suppress the shuttle effect by blocking the migration paths of polysulfides. This not only helps alleviate certain issues in cathode applications but also yields additional positive effects, including but not limited to enhancing long‐term cycling performance and suppressing lithium dendrite growth.^[^ ^141^ ^]^ Furthermore, extensive research has confirmed that when TMSs are employed in the cathode, certain typical issues remain unavoidable during long‐term cycling, which has further driven innovations in the application modes of TMSs. First, the relatively high mass density and limited porosity of sulfide catalysts reduce the energy density of Li–S batteries while simultaneously impeding the attainment of high sulfur loading. Second, the repeated deposition and incomplete dissociation of Li_2_S lead to progressive surface coverage of the cathode catalysts. This results in the passivation of active sites, thereby diminishing catalytic and adsorption capabilities and ultimately causing battery failure. Lastly, the continuous oxidation‐reduction processes of lithium ions during charge–discharge cycles induce dendritic lithium growth on the anode surface. Prolonged cycling allows these dendrites to pierce the separator, leading to internal short circuits and safety hazards. Significantly, the sulfide catalysts located in the cathode compartment cannot mitigate this anode‐related issue, highlighting a critical unresolved challenge. Therefore, the use of sulfides as modifying materials for separators has also been applied to lithium sulfur batteries. As illustrated in **Figure** **14**a, incorporating sulfide‐modified separators into Li–S batteries enables the employment of high porosity host materials or even pure sulfur as cathode components, thereby enhancing active material loading capacity. Fortunately, separators modified with sulfide catalysts (MoS_2_‐SnS) effectively mitigate polysulfides crossover from the cathode side, minimizing the loss of active sulfur species.^[^ ^142^ ^]^

### 3.2 TMSs for Separator Coating

#### 3.2.1 The Structural Integrity of the Catalyst

In contrast to cathode hosts with limited active sites, the sulfide catalysts on the separator provide additional reaction interfaces to accelerate sulfur redox kinetics and reduce polarization phenomena, thereby improving overall electrochemical performance. Furthermore, it is particularly noteworthy that the addition of a minimal quantity of the sulfide catalyst to the separator can achieve highly satisfactory outcomes. This strategic approach ensures that the mass of each component within the battery system can be meticulously controlled within a predefined threshold. Consequently, the reduction in the theoretical energy density of the battery remains within an acceptable range, thereby preserving the overall efficiency and performance of the LSBs. Collectively, these attributes of modified separators offer a multifaceted solution to address the challenges outlined above.

A stable catalyst architecture serves as a prerequisite for attaining high‐efficiency electrocatalysis and enduring cycling stability. To achieve synergistic catalytic performance, it is essential that the active sites of the catalyst preserve their structural integrity and regenerability under cyclic operation conditions. Initially, Shen et al.^[^ ^71^ ^]^ demonstrated that cobalt doping enhanced the electrocatalytic activity of cubic ZnS toward polysulfides (Co_0.125_Zn_0.875_S), while also identifying catalyst passivation phenomena. Afterward, Men et al.^[^ ^143^ ^]^ developed a 10% cobalt doped pyrite‐type iron disulfide (Co_0.1_Fe_0.9_S_2_) electrocatalyst deposited on acetylene black as a separator coating for Li–S batteries. The XPS spectra of this catalyst after interaction with Li_2_S_6_ reveal attenuated SO_4_^2−^ peak intensity (Figure 14b), indicating reduced iron oxide and sulfate content and suppressed surface oxidation of FeS_2_ following cobalt doping. This structural modification effectively prevents deactivation of the FeS_2_ electrocatalyst. Consequently, the battery incorporating AB/Co_0.1_Fe_0.9_S_2_ exhibited excellent cycling performance. At a current density of 1 C, it delivered an initial discharge capacity of 854.7 mAh g^−1^ and maintained stable operation over 1000 cycles without significant capacity decay, as demonstrated in Figure 14c. The construction of composite materials can also leverage the complementary properties of multiple components to achieve stable and enhanced electrochemical performance. Jiao et al.^[^ ^144^ ^]^ designed a self‐healing ZnIn_2_S_4_‐In_2_O_3_‐ZnIn_2_S_4_ catalyst coated on separator surfaces. By assembling ZnIn_2_S_4_ nanosheets on both inner and outer surfaces of In_2_O_3_ nanotubes, a sandwich‐like heterostructure was formed (Figure 14d). Compared to pristine In_2_O_3_ nanosheets (Figure 14e and Figure 14f), the incorporation of ZnIn_2_S_4_ mitigated polysulfide aggregation on the nanotube surfaces while accelerating the conversion of long‐chain Li_2_S_6_/Li_2_S_4_ to short‐chain Li_2_S_2_/L_2_S intermediates, with the formation of LiInS_2_ as a key intermediate product. This catalytic configuration enabled adsorption sites to re‐expose, restoring the catalyst surface during subsequent cycles (Figure 14g), which endows the catalyst with self‐healing capabilities and effectively maintains its structural stability. Notably, the In_2_O_3_‐ZnIn_2_S_4_ battery retained a high specific capacity of 429.5 mAh g^−1^ after 700 cycles at a current density of 1.0 C.

Through a combination of theoretical and computational analysis with experimental validation, Lei et al.^[^ ^145^ ^]^ demonstrated that uniformly distributed oxygen doping on MoS_2_ substrates is able to generate abundant adsorption/catalytic sites and expand the interlayer spacing to facilitate ion transport, while enhancing the catalytic conversion kinetics of polysulfides. Subsequently, they developed a facile ethylene glycol competitive reduction strategy to anchor oxygen‐doped molybdenum disulfide (O‐MoS_2_) onto carbon nanosheets (CNS), yielding a novel composite material for separator functional modification. The modified battery delivered an initial discharge capacity of 1537 mAh g^−1^ at 0.2 C. Remarkably, after 2000 ultra‐long cycles at a high current density of 1 C, the battery still retained a high reversible specific capacity of 545 mAh g^−1^. Furthermore, the properties of sulfides can be readily manipulated through ion doping techniques, which holds significant potential for accelerating polysulfides conversion in LSBs. These outstanding long‐term cycling stabilities further validate that the sulfide catalyst deployed at the separator interface exhibits robust anti‐passivation properties. By effectively preventing passivation, the catalyst sustains its structural integrity. As a result, the Li–S batteries showcase exceptional electrochemical performance.

#### 3.2.2 2D Deposition of Li_2_S

**Figure 15:**
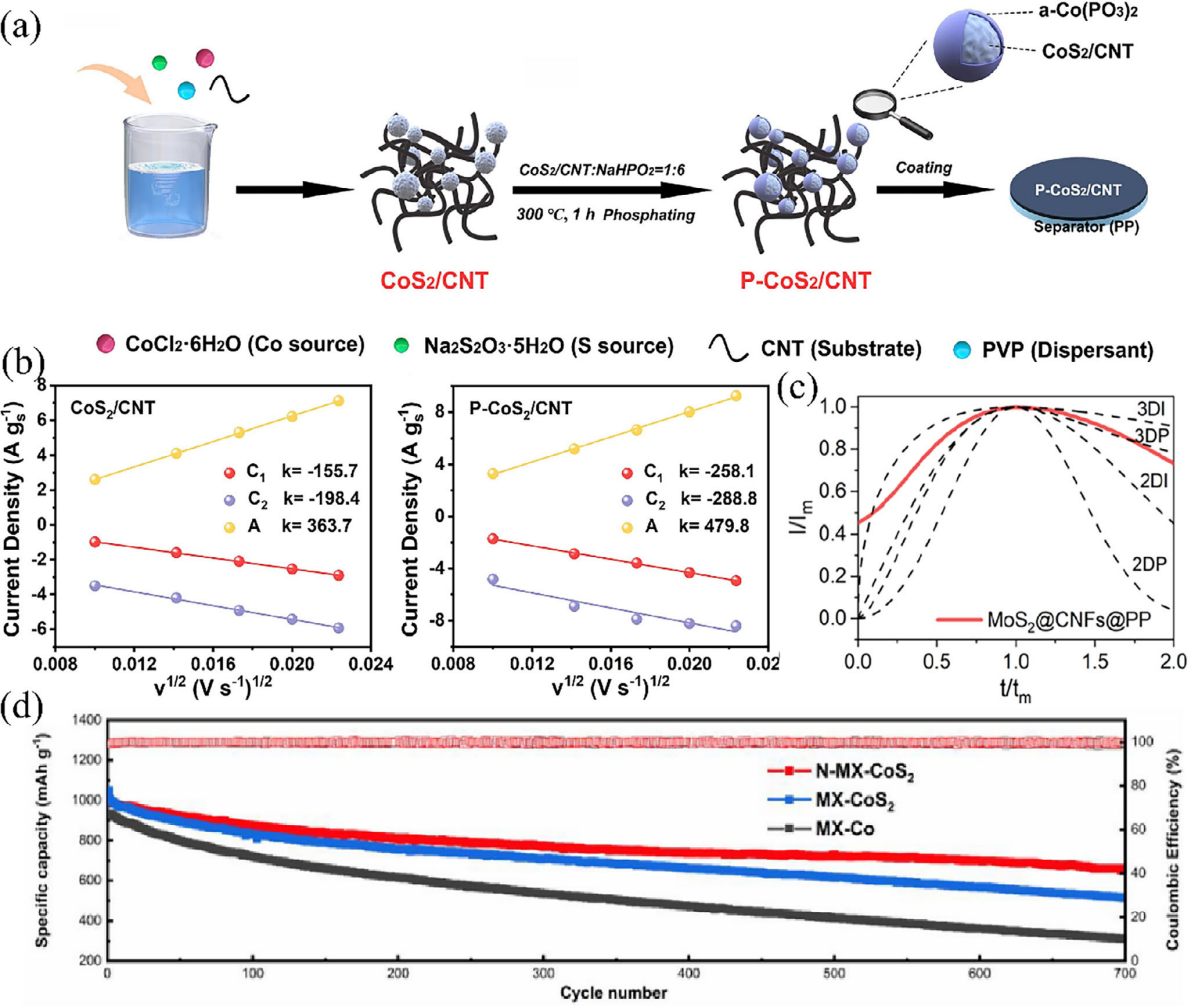
a) Schematic diagram of the synthetic procedures of P‐CoS_2_/CNT and its functional separator. Reproduced with permission.^[^ ^146^ ^]^ Copyright 2024, Elsevier. b) Corresponding linear fits of the peak current densities of CoS_2_/CNT and P‐CoS_2_/CNT. Reproduced with permission.^[^ ^146^ ^]^ Copyright 2024, Elsevier. c) Comparison of dimensionless transient and theoretical models for MoS_2_@CNFs@PP separators. Reproduced with permission.^[^ ^150^ ^]^ Copyright 2024, Elsevier. d) Longterm cycling stability tested at 1.0 C with the three samples. Reproduced with permission.^[^ ^151^ ^]^ Copyright 2022, Elsevier.

Moreover, rationally designed composite materials can effectively minimize the adverse effects of separator coatings on lithium‐ion transport dynamics. By constructing fast ion‐conduction pathways within the separator modification layer, these composites prevent the formation of high‐energy diffusion barriers. Taking a representative example, Zhang et al.^[^ ^146^ ^]^ exploited the excellent electrical conductivity of CoS_2_ (6.7 × 10^3^ S cm^−1^) as a model material. Through a facile and precisely controlled phosphorus regulation strategy (as illustrated in **Figure** **15**a), they fabricated a carbon nanotube‐decorated crystalline‐amorphous heterostructured cobalt disulfide catalyst (denoted as P‐CoS_2_/CNT) for long‐life Li–S cells. This unique heterostructure features a crystalline CoS_2_ core encapsulated within an amorphous Co(PO_3_)_2_ shell. Within this composite, the highly conductive crystalline CoS_2_ core facilitates rapid electron transfer for the heterogeneous catalyst. Meanwhile, the outer amorphous Co(PO_3_)_2_ layer acts as the true catalytic shell. It exposes a rich array of active Co‐O‐P bonds, effectively optimizing the kinetics of polysulfides conversion. Consequently, when P‐CoS_2_/CNT is utilized as a multifunctional interlayer on the polypropylene (PP) separator, this innovative material exhibits outstanding electrochemical performance. The lithium‐ion diffusion rate is determined by analyzing cyclic voltammetry (CV) curves at various scan rates using the well‐established Randles–Sevcik equation.^[^ ^147^ ^]^ As depicted in Figure 15b, the fitting curve of P‐CoS_2_/CNT features the steepest slope (k), which strongly suggests that the P‐CoS_2_/CNT modified separator promotes efficient lithium‐ion transport, thereby accelerating the conversion and decomposition of LiPSs. When the P‐CoS_2_/CNT separator is incorporated, the battery can deliver a more favorable initial specific capacity of 1301.7 mAh g^−1^, significantly outperforming the specific capacity of batteries using other separators. Drawing on the interface engineering strategies, Wang et al.^[^ ^148^ ^]^ achieved precise modulation of the surface and interfacial electronic configurations of transition metal sulfides, which significantly enhanced the adsorption affinity toward lithium polysulfides and expedited the kinetics of sulfur redox reactions. Employing a one‐step hydrothermal synthesis, they successfully fabricated a 3D flower‐shaped NiS_2_‐WS_2_ heterostructure. Comprising nanosheets ≈10 nm in size, this distinctive architecture offers an interconnected network of channels that facilitate rapid Li^+^ diffusion and promote efficient electrolyte infiltration. Therefore, the NiS_2_‐WS_2_ cells delivered an initial specific discharge capacity of 1518.7 mAh g^−1^ at a rate of 0.2 C. Even at a high rate of 5 C, it still achieved an initial specific discharge capacity of 615 mAh g^−1^ and the specific capacity remained at 258.9 mAh g^−1^ after 1500 cycles, with a capacity decay of 0.039% per cycle.

Beyond the previously discussed strategies, manipulating the deposition behavior of lithium sulfide (Li_2_S) and fostering its vertical growth on the sulfide surface emerges as a promising avenue for sustaining the catalyst's high‐level activity. Leveraging the theoretical formula of the current‐time transient, it is possible to delineate four distinct deposition patterns of lithium sulfide, including 2D instantaneous nucleation (2DI), 2D progressive nucleation (2DP), 3D instantaneous nucleation (3DI), and 3D progressive nucleation (3DP).^[^ ^149^ ^]^ Notably, the 2D planar deposition pattern poses a challenge as it has a propensity to blanket the material surface, leading to the deactivation of the catalyst. Conversely, the 3D nucleation pattern offers a distinct advantage by facilitating the exposure of a greater number of adsorption and catalytic sites.

For instance, Liu et al.^[^ ^150^ ^]^ proposed a functional separator modified with flower‐shaped molybdenum sulfide grown on carbon nanofibers and revealed its actual functional state and catalytic mechanism. The fitting curves in Figure 15c clearly indicate that for the MoS_2_@CNFs@PP modified separator, the growth pattern of Li_2_S can be entirely shifted to the 3DP mode, which prevents the deactivation of the catalyst. In addition, the growth rate of Li_2_S is primarily governed by ion diffusion. The swift diffusion of ions across the substrate of surface MoS_2_@CNFs@PP serves as a safeguard against the surface passivation of the sulfur electrode and enables a higher sulfur loading capability. This is because the in situ formed Mo_2_S_3_/MoS_2_@CNFs composite on the separator during the cycling process can boost the electron transfer between sulfur species and MoS_2_. The emergence of Mo_2_S_3_ serves a dual‐purpose function. First, it acts as a protective shield for the inner MoS_2_ catalyst, effectively preserving the structural integrity of the composite, which is crucial for its long‐term performance. Second, Mo_2_S_3_ offers a novel reduction pathway for the dissolved polysulfides. This newly‐established pathway streamlines the chemical reactions involving sulfur species, enabling a more efficient reversible transformation of sulfur. Consequently, the LSBs incorporating the MoS_2_@CNFs@PP separator have achieved an areal capacity of 5.8 mAh cm^−2^ under a high sulfur loading condition of 4.5 mg cm^−2^. Specifically, they exhibited an initial specific discharge capacity of 839.5 mAh g^−1^ at a current rate of 1 C. Even after 500 cycles, the battery managed to preserve 58.6% of its initial capacity, thereby manifesting remarkable stability in long‐term cycling performance. Analogously, Yang and colleagues employed a one‐step in situ sulfuration approach to engineer a novel N‐doped MXene‐CoS_2_ nanohybrid (NMX‐CoS_2_).^[^ ^151^ ^]^ The synergistic interplay of chemical adsorption between the nitrogen‐doped sites and the polar CoS_2_ nanoparticles endows this nanohybrid with a remarkable affinity for LiPSs, ensuring 3D deposition of Li_2_S. The MXene substrate, characterized by an expanded layered structure, possesses exceptional electrical conductivity, which serves as a catalyst for facilitating rapid electrochemical redox reactions of sulfur, thereby expediting the reaction kinetics. The batteries with the N‐doped MXene‐CoS_2_ modified separator exhibit a high initial specific capacity of 1031 mAh g^−1^ at a rate of 1 C and maintains a stable capacity decay rate over 700 cycles, as shown in Figure 15d.

The cycling performance of LSBs with separator coating is summarized in **Table** **3**. Compared with the application of modification on the cathode, this modification method significantly improves the ultra‐long cycling performance and sulfur loading of the batteries.

**Table 3:** The summary of the cycling performance of LSBs with modified separators.

Catalysts	Sulfur loading [%]	Current density [C]	Initial‐capacity [mAh g^−1^]	Cycles numbers	Capacity retention [%]	Refs.
AB/Co_0.1_Fe_0.9_S_2_	56	1	854.7	920	51.57	[143]
In_2_O_3_‐ZnIn_2_S_4_	72	1	907.4	700	47.35	[144]
O‐MoS_2_/CNS	75	1	1036.12	2000	52.6	[145]
P‐CoS_2_/CNT	72	2	910.4	1000	51.58	[146]
NiS_2_‐WS_2_	70	5	615	1500	42.1	[148]
MoS_2_@CNFs@PP	70	1	839.5	500	58.6	[150]
NMX‐CoS_2_	80	1	1031	700	63.14	[151]
MoS_2_@MGF	56	1	985	2000	47	[152]
ZnS‐FeS/HPC	‐	3	847.3	1500	34	[153]

In summary, this chapter focuses on elucidating the effects of TMSs in the separator on the sulfur cathode, including enhancing the cathode's specific discharge capacity and enabling long‐term cycling performance of the battery. However, the impact of this application mode on battery components goes further; it also encompasses effects on the anode. For instance, the coated separator can facilitate uniform transport of lithium ions, enabling uniform deposition on the anode surface, thus suppressing lithium dendrite growth. As is widely recognized, the stability of the anode directly dictates the overall performance of the battery. Therefore, modification of the lithium anode is also a necessary and effective measure to enhance the performance of LSBs.

## 4 TMSs for Dendrite‐Free Lithium Anode

Although the two aforementioned application strategies for TMSs have both contributed to enhancing the performance of LSBs, during cycling, the continuous deintercalation and deposition of lithium ions trigger uncontrollable lithium dendrite growth on the anode surface—an issue that significantly impedes further improvements to other key LSB performances. Therefore, the development of dendrite‐free lithium anodes serves as a core pillar for advancing the technological iteration and upgrading of LSBs, as well as ultimately enabling their commercialization. To date, researchers have proposed numerous strategies to suppress dendrite formation on lithium anodes, including: introducing functional interlayers between the separator and anode, utilizing lithium hosts or lithium alloys as alternatives to traditional metallic lithium anodes, and adopting solid‐state electrolytes. Among these approaches, TMS catalysts, leveraging their unique interface modulation capabilities, are frequently engineered into separator coatings or lithium host matrices. This design enables the achievement of dendrite‐free lithium anodes by modulating the uniform transport and deposition kinetics of lithium ions, while also delivering a notable synergistic boost to the comprehensive performance of LSBs.

### 4.1 Promote the Uniform Transport of Lithium Ions

**Figure 16:**
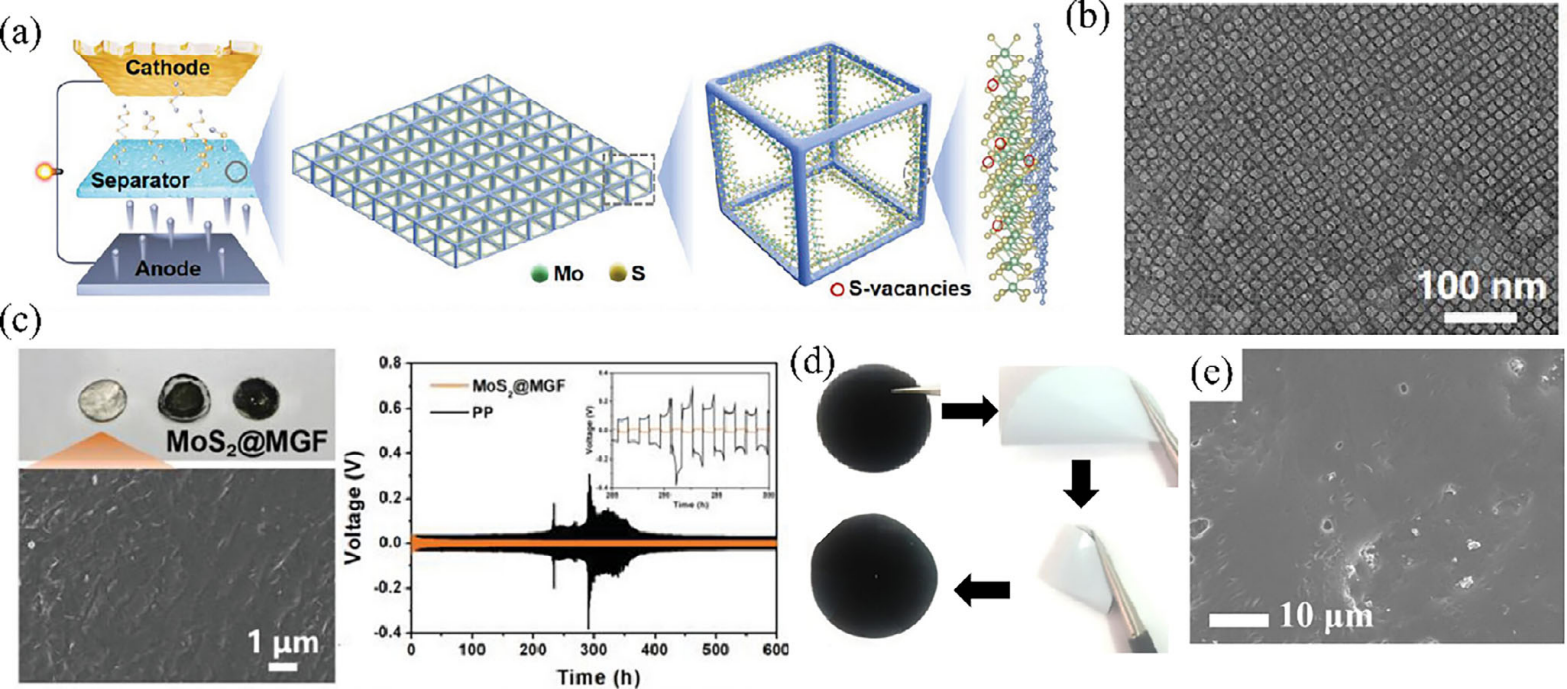
a) Schematic of MoS_2_@MGF as an interlayer for Li–S batteries. Reproduced with permission.^[^ ^152^ ^]^ Copyright 2024, Wiley‐VCH. b) TEM images of MoS_2_@MGF. Reproduced with permission.^[^ ^152^ ^]^ Copyright 2024, Wiley‐VCH. c) Voltage profiles of Li||Li symmetric cells at 1 mA cm^−2^ and 1 mAh cm^−2^ with MoS_2_@MGF and photographs of lithium electrodes and separators in Li||Li symmetric after 600 h of cycling. Reproduced with permission.^[^ ^152^ ^]^ Copyright 2024, Wiley‐VCH. d) Digital photos of ZnS‐FeS/HPC‐PP (The process of folding and unfolding a separator). Reproduced with permission.^[^ ^153^ ^]^ Copyright 2025, Elsevier. e) Top‐view SEM images at 10 nm of ZnS‐FeS/HPC‐PP. Reproduced with permission.^[^ ^153^ ^]^ Copyright 2025, Elsevier.

The sulfide catalyst located on the side of the separator adjacent to the lithium anode cannot only block the shuttling of polysulfides but also make the deposition of lithium ions more uniform, thereby reducing the formation of lithium dendrites. Consequently, these advantageous effects culminate in the realization of a LSB endowed with both long‐term cycling stability and enhanced safety features. To illustrate this concept, Gao and colleagues employed a template‐based synthesis approach to fabricate a 2D film.^[^ ^152^ ^]^ This innovative film features a unique architecture where single‐layer cubic graphite cages enclose few‐layer curved MoS_2_. This unique 2D structure is realized through a precise process of confining the epitaxial growth of MoS_2_ within the open cages of a 2D ordered mesoporous graphite framework (MGF). During this process, the growth of MoS_2_ is restricted by the well‐defined environment of the MGF, which acts as a template guiding the formation of the structure. Consequently, the MoS_2_@MGF heterostructure featuring a high density of sulfur vacancies is successfully fabricated (**Figure** **16**a). And the ordered mesoporous structure and excellent electrical conductivity of MGFs can facilitate the exceptionally uniform and comprehensively extensive migration of lithium ions (Figure 16b). As a result, it becomes possible to simultaneously realize a sulfur cathode free from the shuttle effect and a lithium anode without dendrite formation. When the separator treated with MoS_2_@MGF is incorporated into a lithium symmetric battery, at a current density of 1 mA cm^−2^, the battery exhibits outstanding stability. It manages to maintain a relatively stable voltage and a low overpotential over a continuous operation period of up to 600 h and the scanning electron microscopy (SEM) images of the lithium foil after cycling also reveal a relatively smooth surface (as shown in Figure 16c). This finding demonstrates that the MoS_2_@MGF composite material can suppress the growth of lithium dendrites and enable long‐cycle operation of the battery.

Analogously, Ren and colleagues engineered a ZnS‐FeS heterostructure embedded within a hierarchically porous carbon matrix (ZnS‐FeS/HPC), which also accelerates the reduction of Li_2_S_2_ and inhibits the growth of lithium dendrites.^[^ ^153^ ^]^ As visually presented in Figure 16d, the separator coated with the ZnS‐FeS/HPC composite showcases remarkable flexibility and robust mechanical strength. These characteristics are of paramount importance as they underpin the stability of the battery during repeated charge–discharge cycles. Furthermore, the ZnS‐FeS/HPC‐PP battery exhibits an outstanding ability to achieve ultrafine and smooth lithium nucleation. This favorable nucleation behavior leads to the establishment of a smooth interface with a significantly reduced presence of dendrites (Figure 16e). The exceptional performance can be ascribed to the intimate interface and the profusion of active sites within the ZnS‐FeS/HPC composite. These features provide an ideal environment for lithium‐ion deposition and reaction, minimizing the formation of dendritic structures. Finally, the ZnS‐FeS/HPC material not only demonstrates a long‐cycle time of nearly 500 h in a symmetric battery but also can stably cycle for 1500 cycles at a high rate of 3 C with an extremely low‐capacity decay rate (0.044%). A well‐conceived design of sulfide structures holds significant promise for direct application in lithium metal anodes. Such designs play a crucial role in precisely regulating the electronic state and facilitating the uniform deposition of lithium ions.

### 4.2 Lithium stabilizers used for the uniform deposition of lithium ions

**Figure 17:**
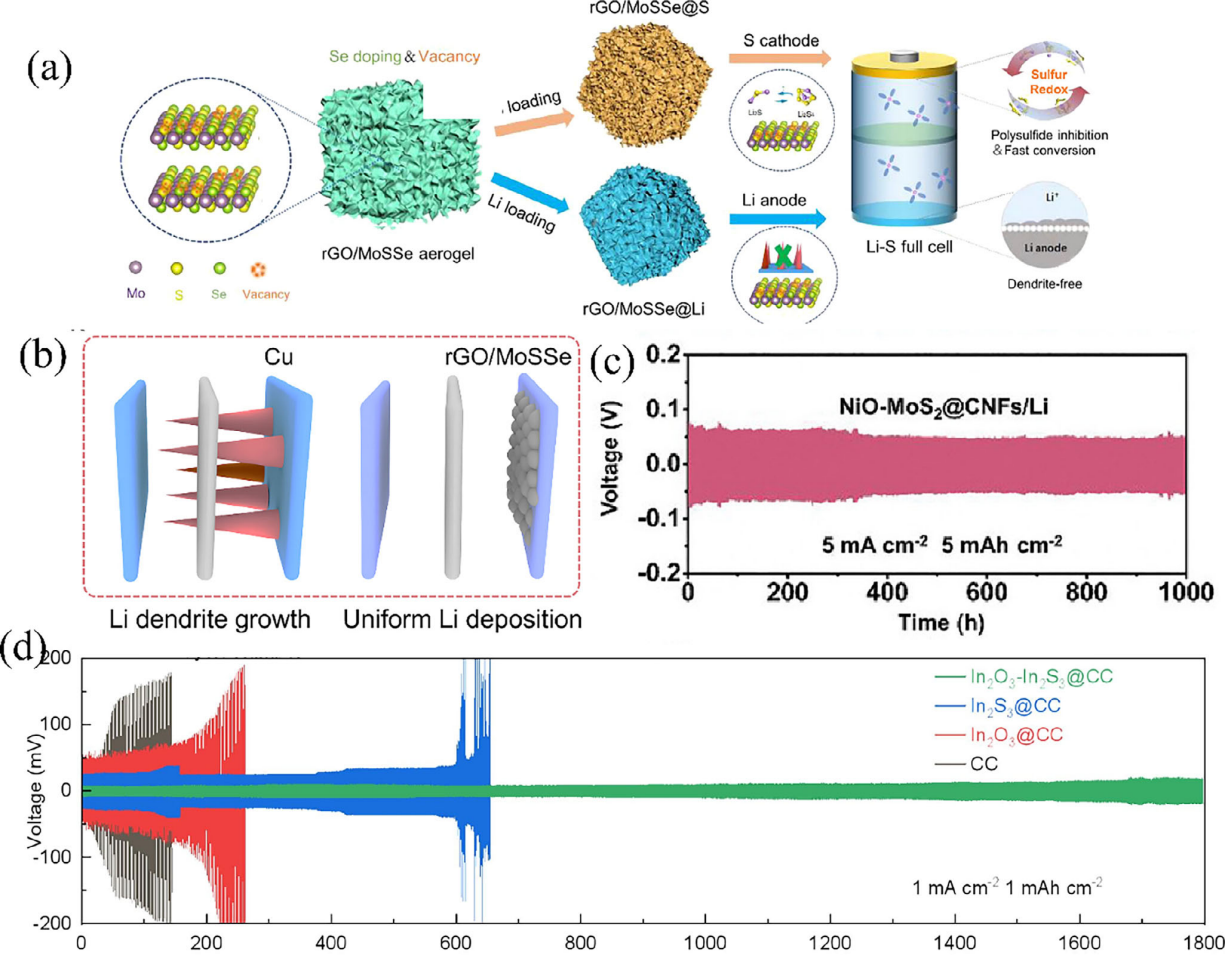
a) Schematic of the sulfur and Li loading processes in a vacancy‐rich rGO/MoSSe aerogel for the cathode and anode of LSBs. Reproduced with permission.^[^ ^154^ ^]^ Copyright 2022, American Chemical Society. b) Schematic illustration of the plating/stripping processes on Cu foil and rGO/MoSSe. Reproduced with permission.^[^ ^154^ ^]^ Copyright 2022, American Chemical Society. c) Electrochemical performances of NiO‐MoS_2_@CNFs anodes in symmetric cells. Reproduced with permission.^[^ ^155^ ^]^ Copyright 2023, Wiley‐VCH. d) Electrochemical performance of In_2_O_3_‐In_2_S_3_@CC/Li||Li symmetric cell at 1 mA cm^−2^/1 mAh cm^−2^. Reproduced with permission.^[^ ^156^ ^]^ Copyright 2025, Elsevier.

To establish stronger interactions with lithium metal, TMS catalysts have been directly applied to the anode side. Functioning as stabilizers for lithium metal, these catalysts effectively suppress the growth of lithium dendrites. For example, in order to enhance the lithiophilicity of MoS_2_ and inhibit the growth of lithium dendrites, Li et al.^[^ ^154^ ^]^ deposited Se‐doped MoS_2_ onto a redox graphene aerogel with high porosity (MoSSe/rGO), which was subsequently used to carry sulfur and lithium as the cathode and anode of the battery, respectively (**Figure** **17**a). The Se within the crystal lattice increases the number of anions and promotes the rapid migration and uniform deposition of lithium ions. Eventually, a dendrite‐free lithium anode is achieved during the operation of the battery (Figure 17b). At a current density of 2 mA cm^−2^, the rGO/MoSSe@Li||Li symmetric battery can still cycle stably for 1200 h while maintaining a small overpotential within 20 mV. The S@rGO/MoSSe||rGO/MoSSe@Li full battery also exhibits excellent cycling stability. After cycling 1000 times at a high rate of 1 C, it can still maintain a high reversible specific capacity of 637.3 mAh g^−1^. These results all indicate that the rGO/MoSSe composite material has a strong ability to suppress the growth of lithium dendrites and restrict the shuttling of polysulfides, ultimately endowing LSBs with excellent electrochemical performance.

In this regard, Du and colleagues embarked on a research endeavor and successfully developed a p‐n type heterostructure.^[^ ^155^ ^]^ This innovative structure is intended to function as a lithium stabilizer for the anode in practical LSBs. This heterostructure is composed of p‐type NiO nanoparticles and n‐type MoS_2_ nanosheets. Due to the difference in their electronic states, an internal built‐in electric field is generated at the interface after the formation of the heterojunction, which can regulate the electron migration. Therefore, it can prevent the growth of lithium dendrites by enhancing the lithiophilicity of the substrate and reducing the local current density. To evaluate the lithiophilicity of the NiO‐MoS_2_@CNFs material, a series of tests were conducted using a lithium foil as the counter‐electrode. Even at a high current density of 5 mA cm^−2^, the NiO‐MoS_2_@CNFs/Li battery still exhibited excellent cycling stability for over 1000 h, as shown in Figure 17c. Furthermore, in the electrochemical impedance spectroscopy (EIS) test, the battery demonstrated an extremely low interfacial resistance of the solid‐electrolyte interphase (SEI) film. The origin of this low‐resistance phenomenon lies in the formation of a stable SEI film at the electrolyte‐anode interface during lithium‐ion deposition/stripping. The film not only functions as a protective barrier to preserve the stability of the electrode structure but also optimizes interfacial contact between the electrolyte and anode, thereby accelerating lithium‐ion transport between them and enhancing the redox kinetics of polysulfides. Furthermore, the built‐in electric field within the NiO‐MoS_2_@CNFs structure facilitates efficient and directional charge transport, ensuring the charge supply demanded by sulfur reactions. Leveraging these advantages, the battery ultimately exhibits low impedance. In conclusion, this study not only showcases the potential of the p‐n type heterostructure but also provides a promising avenue for the development of high‐performance LSBs in the future.

In a parallel line of research, Li et al.^[^ ^156^ ^]^ adopted a straightforward hydrothermal route to achieve in situ growth of In_2_O_3_‐In_2_S_3_ heterostructures on carbon cloth. The as‐synthesized In_2_O_3_‐In_2_S_3_@CC composite features outstanding sulfurphilicity and lithiophilicity properties. Such properties are of paramount importance in the pursuit of reliable Li–S batteries. To probe the efficacy of the heterostructure in maintaining stability, lithium was deposited onto the In_2_O_3_‐In_2_S_3_@CC composite, which functioned as the anode component of the battery. The interface between the two semiconductors gives rise to a BIEF. This field serves as a driving force for the uniform transfer of electrons and lithium ions, impeding the formation and growth of lithium dendrites. By preventing dendrite‐related failures, the In_2_O_3_‐In_2_S_3_@CC/Li anode endows batteries with enhanced cycling stability, thus providing a promising solution for the practical application of LSBs. At a current density of 1 mA cm^−1^, the Li||Li symmetric battery with In_2_O_3_‐In_2_S_3_@CC/Li serving as the anode can achieve stable cycling for 1800 h. Meanwhile, its overpotential remains below 10 mV, which indirectly demonstrates that the catalyst possesses a remarkable capacity to regulate lithium dendrites (Figure 17d).

Finally, the potentials of a series of symmetric cells and the corresponding cycle times under galvanostatic conditions are summarized in **Table** **4**. These results demonstrate that TMSs catalysts can significantly suppress the growth of lithium dendrites, enabling safer and more efficient LSBs.

**Table 4:** Cycling time and overpotential of symmetric cells under galvanostatic conditions.

Catalysts	Current density [mA cm^−1^]	Capacity [mAh cm^−1^]	Cycles time [h]	Over potential [mV]	Refs.
MoS_2_@MGF	1	1	600	36	[152]
ZnS‐FeS	1	1	500	11	[153]
MoSSe/rGO	2	2	1200	20	[154]
NiO‐MoS_2_	10	10	1000	79	[155]
In_2_O_3_‐In_2_S_3_	1	1	1800	10	[156]

In summary, TMS catalysts demonstrate remarkable efficacy in stabilizing lithium anodes and enhancing safety. The properties of TMSs and other representative anode materials are summarized in **Table** **5**. By comparing the capacity, lithium storage mechanism, and cost of various materials, it is evident that TMSs, as anode materials, exhibit higher capacity, superior structural and chemical stability, and lower cost—rendering them anode materials with broader application prospects. Their applications are not limited to lithium battery systems but also extend to fields such as sodium/potassium‐sulfur batteries, zinc batteries, and aluminum batteries. However, despite the application of TMSs on the lithium anode aiding in inhibiting lithium dendrite growth, notable limitations persist: some polysulfides inevitably shuttle to the anode, where they undergo catalyst‐driven conversion reactions with the lithium anode, potentially triggering micro‐short circuits. Although this phenomenon is relatively likely to occur, there remains a lack of adequate research to support this conclusion. Therefore, future exploration into the pros and cons of TMSs in lithium anodes will constitute a valuable research avenue. However, given the complexity of battery systems, this endeavor not only calls for more advanced characterization techniques but also demands the wisdom and patience of researchers. Furthermore, certain catalysts react directly with lithium metal during long‐term charge–discharge cycling, resulting in severe self‐discharge. Thus, performance optimization of LSBs cannot be confined to a single component. Instead, it must encompass synergistic optimization across multiple dimensions—cathode, separator, and anode—alongside the selection of appropriate TMS catalysts. Only through such an approach can the impact of various adverse factors be minimized, ultimately enabling the battery to achieve high performance. Additionally, hampered by the complex preparation processes and high costs of Li/TMS composites, large‐scale, low‐cost production routes remain to be further explored and optimized.

**Table 5:** Summary of properties of several typical anode materials.

Anode	Capacity [mAh g^−1^]	Pontential [V]	Working mechanism	Volume expansion ratio	Cost	Refs.
Titanium‐based oxides (TiO_2_, Li_4_Ti_5_O_12_)	175–300	1.4–1.7	Ion conversion reaction	≈5%	Medium	[157]
Graphite	300–400	0.05–0.2	Ion intercalation and deintercalation	≈10%	Low	[158]
Hard carbons	450–600	0.1–0.3	Ion intercalation and deintercalation	≈20%	Low	[158]
TMSs (MoS_2_, CoS_2_, SnS_2_)	500–1000	0.6–1	Ion conversion reaction	≈100%	Medium	[159]
Graphene	300–600	0.2–0.4	Ion intercalation and deintercalation	≈10%	High	[158]
Si	3579	0.1–0.5	Alloying reaction	≈300%	Medium	[160]
Alloys (Li/Al, Li/Zn, Li/Ag)	500–1000	0.4–0.6	Alloying reaction	≈420%	High	[157]
Alloys (Li/Si, Li/Sn, Li/Ge)	1000–3000	0.3–0.6	Alloying reaction	≈420%	High	[157]
Phosphorus	2596	0.9–1.1	Alloying reaction	≈300%	Medium	[161]
Li metal	3860	0–0.1	Deposition and dissolution reactions	≈300%	High	[162]

## 5. Conclusion and Perspectives

This review elaborates on the applications of TMS catalysts in the cathode, the separator, and the anode of LSBs. Numerous studies have shown that the application of TMSs in LSBs can mitigate LSBs’ inherent drawbacks and enhance performance in multiple aspects, including but not limited to improving the discharge specific capacity, enhancing long‐cycle stability, and optimizing safety performance. Furthermore, TMSs help reduce the proportion of electrolyte dosage, thereby mitigating the dissolution and shuttle effect of polysulfides. This enables LSBs to retain high capacity and cycling stability even under low electrolyte/sulfur ratios (4–6 µL mg^−1^).^[^ ^163^, ^164^ ^]^ However, while at the laboratory scale, TMSs have shown significant enhancing effects, enabling LSBs to deliver excellent performance, their performance still falls short in pouch cells and larger‐scale deployment. This is primarily because higher sulfur content exacerbates the shuttle effect, and with increasing electrode size, the efficacy of TMSs drops significantly. Additionally, in contrast to carbon materials, TMSs exhibit higher mass density, inevitably lowering the battery's energy density.^[^ ^165^ ^]^ Thus, future research should focus on the following pressing issues to be resolved and promising future research directions.

1) For the cathode, doped TMSs can increase the initial specific capacity, but they have little effect on improving the cycling performance and rate performance. Therefore, in subsequent research, suitable types of doping elements and their proportions should be explored to ensure stronger stability of the crystal structure. Multi‐metal sulfides and heterostructures can exhibit excellent performance under high rates and long cycling conditions while maintaining a relatively high initial specific capacity. However, due to the complexity of the components, it is difficult to explore the reaction mechanism and develop new efficient catalysts, especially in the design of high‐entropy TMSs and multi‐component heterostructures. Leveraging high‐throughput computational methods to investigate the properties of TMSs enables precise identification of suitable elements. By integrating these computational insights with experimental research, the complex exploration process is significantly streamlined, accelerating the development of highly efficient TMS catalysts. For novel materials such as MOFs and MXenes, two key aspects merit prioritization, exploring more environmentally friendly synthesis methods and modifying the material surfaces to form tighter integration with TMSs.
2) Separators coated with TMSs typically enable LSBs to achieve ultra‐long cycling exceeding 1000 cycles by interrupting the shuttling pathways of polysulfides and preventing catalyst passivation. However, the easy detachment of TMS slurries and their uneven thickness hinder lithium‐ion migration. Additionally, this performance can only be realized in laboratory‐scale coin cells, with limited feasibility for practical applications. For pouch cells, the internal active material content and separator area are far higher than those of coin cell separators, exacerbating the shuttling effect. Therefore, improving separator coating methods and optimizing the types and dosages of binders and conductive agents represent effective approaches to promote the development of Li–S pouch batteries.
3) TMSs promote uniform deposition of lithium ions. When used in lithium anodes, they significantly inhibit the growth of lithium dendrites, thereby greatly enhancing safety performance. Meanwhile, they also facilitate the formation of the SEI film and improve the interfacial state, ensuring the long‐term cycling stability of LSBs. However, the expansion caused by lithium ions intercalation leads to structural damage of TMSs. Researchers often employ electroplating processes to prepare TMS/lithium composite anodes, which suffer from complex procedures, high energy consumption, and difficulties in large‐scale application. Therefore, future research should focus more on the structural design of TMSs (such as porous or core‐shell structures) to ensure stable lithium loading. Additionally, developing simpler and more environmentally friendly preparation methods for Li/TMS composite anodes is also a way to promote the development of LSBs.

TMS catalysts exhibit enormous application potential. By synthesizing the advantages and disadvantages of the three modification methods mentioned above, applying TMS catalysts to the cathode, separator, and anode simultaneously is regarded as an effective approach to achieve high‐performance LSBs. However, the relatively large mass of TMSs leads to a significant reduction in battery energy density. Therefore, research on this type of catalyst needs to focus on component design, structural regulation, and performance optimization to develop more efficient nanoscale TMS catalysts. Specific strategies include the combination of single‐atom catalysts with TMSs, high‐entropy TMSs, and the construction of multi‐component heterostructures. Furthermore, efforts should be directed toward improving the preparation processes of TMSs. Currently, mainstream synthesis methods include hydrothermal method, solid‐phase sintering, and sulfidation reaction, among others. However, such approaches are limited in their ability to precisely regulate the microscale size and elemental distribution of the materials. Therefore, emphasis should be placed on adopting preparation techniques like chemical vapor deposition, which, through regulating the atomic species, proportions, and distribution of transition metals, can further enhance interactions between different atoms, thereby yielding high‐performance and stable TMS catalysts. Additionally, the liquid electrolyte is one of the main causes of the shuttling effect, and solid‐state electrolytes with absolute barrier properties against polysulfides are considered potential solutions to the challenges of Li–S batteries. Introducing TMSs into electrolytes or solid‐state electrolytes to induce the formation of stable SEI films is also a highly promising research direction. We believe that with the continuous advancement and accumulation of the above research, the development of LSBs will surely usher in new breakthroughs.

## Conflict of Interest

The authors declare no conflict of interest.
